# Tissue tropism and transmission ecology predict virulence of human RNA viruses

**DOI:** 10.1371/journal.pbio.3000206

**Published:** 2019-11-26

**Authors:** Liam Brierley, Amy B. Pedersen, Mark E. J. Woolhouse

**Affiliations:** Centre for Immunity, Infection and Evolution, Institute of Evolutionary Biology, University of Edinburgh, Edinburgh, United Kingdom; Princeton University, UNITED STATES

## Abstract

Novel infectious diseases continue to emerge within human populations. Predictive studies have begun to identify pathogen traits associated with emergence. However, emerging pathogens vary widely in virulence, a key determinant of their ultimate risk to public health. Here, we use structured literature searches to review the virulence of each of the 214 known human-infective RNA virus species. We then use a machine learning framework to determine whether viral virulence can be predicted by ecological traits, including human-to-human transmissibility, transmission routes, tissue tropisms, and host range. Using severity of clinical disease as a measurement of virulence, we identified potential risk factors using predictive classification tree and random forest ensemble models. The random forest approach predicted literature-assigned disease severity of test data with mean accuracy of 89.4% compared to a null accuracy of 74.2%. In addition to viral taxonomy, the ability to cause systemic infection was the strongest predictor of severe disease. Further notable predictors of severe disease included having neural and/or renal tropism, direct contact or respiratory transmission, and limited (0 < R_0_ ≤ 1) human-to-human transmissibility. We present a novel, to our knowledge, comparative perspective on the virulence of all currently known human RNA virus species. The risk factors identified may provide novel perspectives in understanding the evolution of virulence and elucidating molecular virulence mechanisms. These risk factors could also improve planning and preparedness in public health strategies as part of a predictive framework for novel human infections.

## Abstract 2

Comparative analysis using machine learning shows that specificity of tissue tropism and transmission biology can act as predictive risk factors for the virulence of human RNA viruses.

## Introduction

The emergence of novel infectious diseases continues to represent a threat to global public health. Emerging pathogens have been defined as those newly recognised infections of humans following zoonotic transmission or those increasing in incidence and/or geographic range [1]. High-profile examples of emerging pathogens include the discovery of the novel Middle East respiratory syndrome (MERS) coronavirus from cases of respiratory illness in 2012 [2] and the expansion of the range of Zika virus across the South Pacific and the Americas [3]. The emergence of previously unseen viruses means that the set of known human viruses continually increases by around two species per year [4,5]. Initial comparative studies identified trends among emerging human pathogens, e.g., increased risk of emergence for pathogens with broad host ranges and RNA viruses [6–9]. However, more recent comparative analyses have focused on risk factors for specific pathogen traits such as transmissibility [10–12]. Here, we focus on understanding the ecological determinants of pathogen virulence, using all currently recognised human RNA viruses as a study system.

Emerging RNA viruses vary widely in their virulence, with some never having been associated with human disease at all. For example, Zaire ebolavirus causes severe haemorrhagic fever with outbreaks, including the 2014 West African outbreak, showing case fatality ratios (CFRs) of approximately 60% or more [13,14]. In contrast, human infections with Reston ebolavirus have never exhibited any evidence of disease symptoms [15]. Applying the comparative approach to understand the ecology of virulence could offer valuable synergy with studies of emergence towards prioritisation and preparedness in the detection of potential new human viruses [16].

Few comparative analyses have addressed the risk factors driving human pathogen virulence to date (but see [17–19]), and none have investigated virulence across the entire breadth of currently recognised human RNA viruses. Of relevance here is an ongoing, largely theoretical debate about the possibility of an evolutionary tradeoff between virulence and transmissibility, which has proven challenging to empirically characterise [20–22]. We also note that in the absence of coevolution, a zoonotic virus may demonstrate ‘coincidental’, nonadapted virulence [23,24]. We therefore compared viruses with different levels of transmissibility in human populations. Transmission route is another potential predictor of virulence; higher mortality rates have been observed in earlier comparative analyses for vector-borne pathogens [17] and pathogens with greater environmental persistence [18]. We therefore hypothesised vector-borne transmission or routes with environmental components (e.g., faecal–oral or food-borne transmission) would be associated with higher virulence than direct, contact-based transmission.

Several studies have suggested a link between host range breadth and virulence, in which higher virulence has been predicted for pathogens with a narrower, specialist host range [25]. Virulence (or host exploitation) has also been predicted to vary with host relatedness through phylogenetic distance [26,27] or in phylogenetic clustering [28]. We therefore hypothesised that a narrow host range, and specifically, infection of nonhuman primate hosts, may also predict virulence. Finally, we hypothesised that a broader tissue tropism could predict higher virulence. This idea is largely unexplored, although experimental studies have demonstrated a broader tissue tropism for more virulent strains of Newcastle disease virus [29].

We aimed to determine patterns of virulence across the breadth of all known human RNA viruses. We then aimed to use predictive machine learning models to ask whether ecological traits of viruses can act as predictive risk factors for virulence in humans. Specifically, we examined hypotheses that viruses would be more highly virulent if they lacked transmissibility within humans, had vector-borne or faecal–oral transmission routes, had a narrow host range or infected nonhuman primates, or had greater breadth of tissue tropisms.

## Results

### Virulence of human RNA viruses

**Fig 1:**
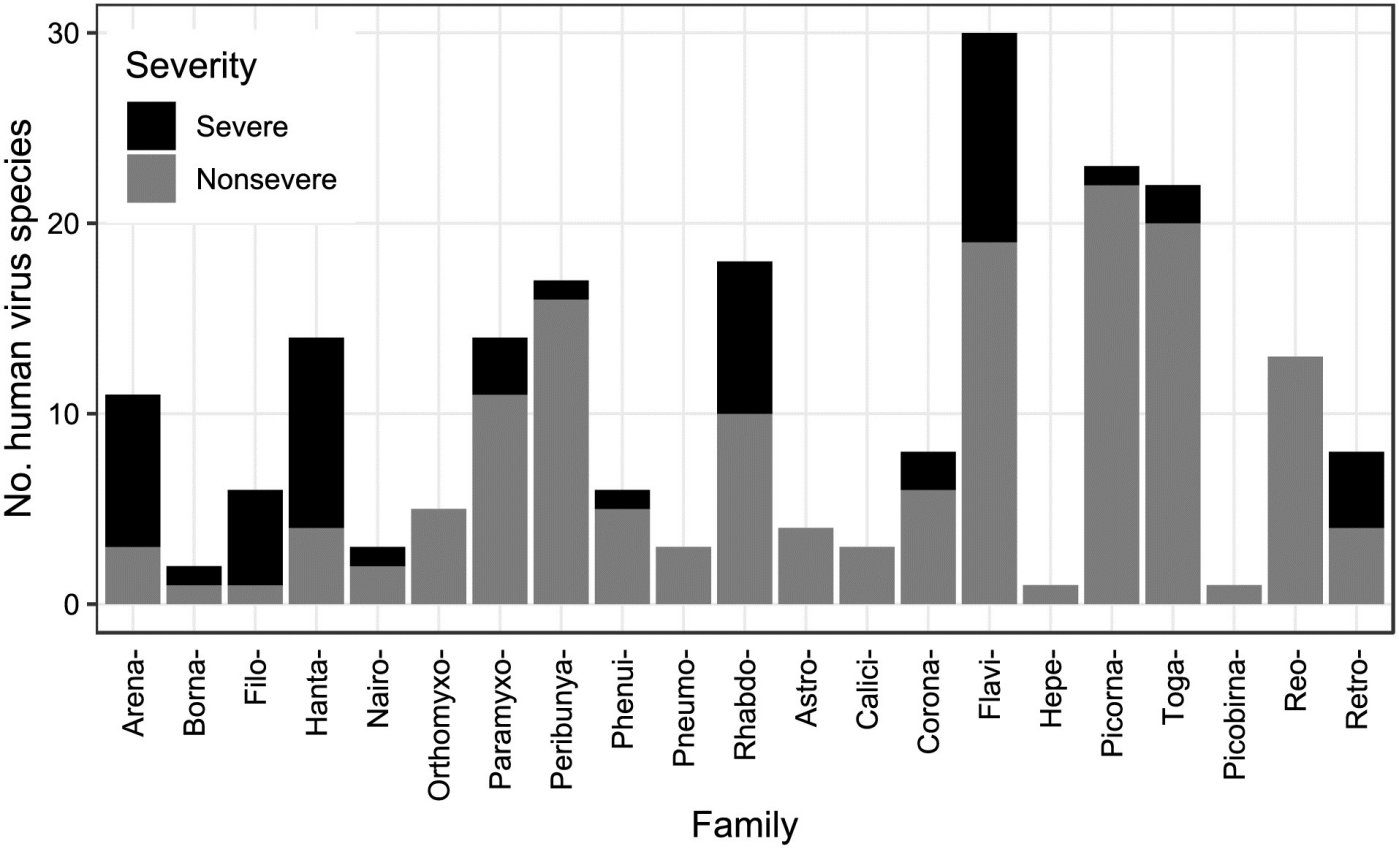
Virulence of currently known human RNA viruses with respect to taxonomy. Number of known human RNA virus species split by ICTV taxonomic family. Shading denotes disease severity rating. Supporting data are available via figshare: 10.6084/m9.figshare.7406441.v3 (https://figshare.com/articles/Data_and_supporting_R_script_for_Tissue_Tropism_and_Transmission_Ecology_Predict_Virulence_of_Human_RNA_Viruses/7406441/3). ICTV, International Committee on Taxonomy of Viruses.

Following [5], as of 2015, there were 214 RNA virus species containing viruses capable of infecting humans, spanning 55 genera and 21 families (with one species unassigned to a family). Using a two-category system, 58 of these were rated as causing ‘severe’ clinical disease and 154 as ‘nonsevere’ following systematic literature review (Fig 1; see also S1 Table). Two viruses could not be assigned a disease severity rating and were excluded from all analyses (hepatitis delta virus, which is reliant on hepatitis B virus coinfection, and primate T-lymphotropic virus 3, which may be associated with chronic disease like other T-lymphotropic viruses but has not been known in humans long enough for cohort observations). Disease severity differed between viral taxonomic families (Fisher’s exact, 1,000 simulations, *p* < 0.001), with Arenaviridae, Filoviridae, and Hantaviridae having the highest fractions of severe-rated virus species (Fig 1). Although 55 of 172 viruses considered zoonotic were rated ‘severe’, we note that only 3 of 40 nonzoonotic viruses were rated as causing severe disease (hepacivirus C and human immunodeficiency virus [HIV] 1 and 2). Fatalities were reported in healthy adults for 64 viruses and in vulnerable individuals only for an additional 26 viruses, whilst eight viruses rated ‘nonsevere’ had severe strains, six of which belonged to the family Picornaviridae.

### Classification tree risk factor analysis

**Fig 2:**
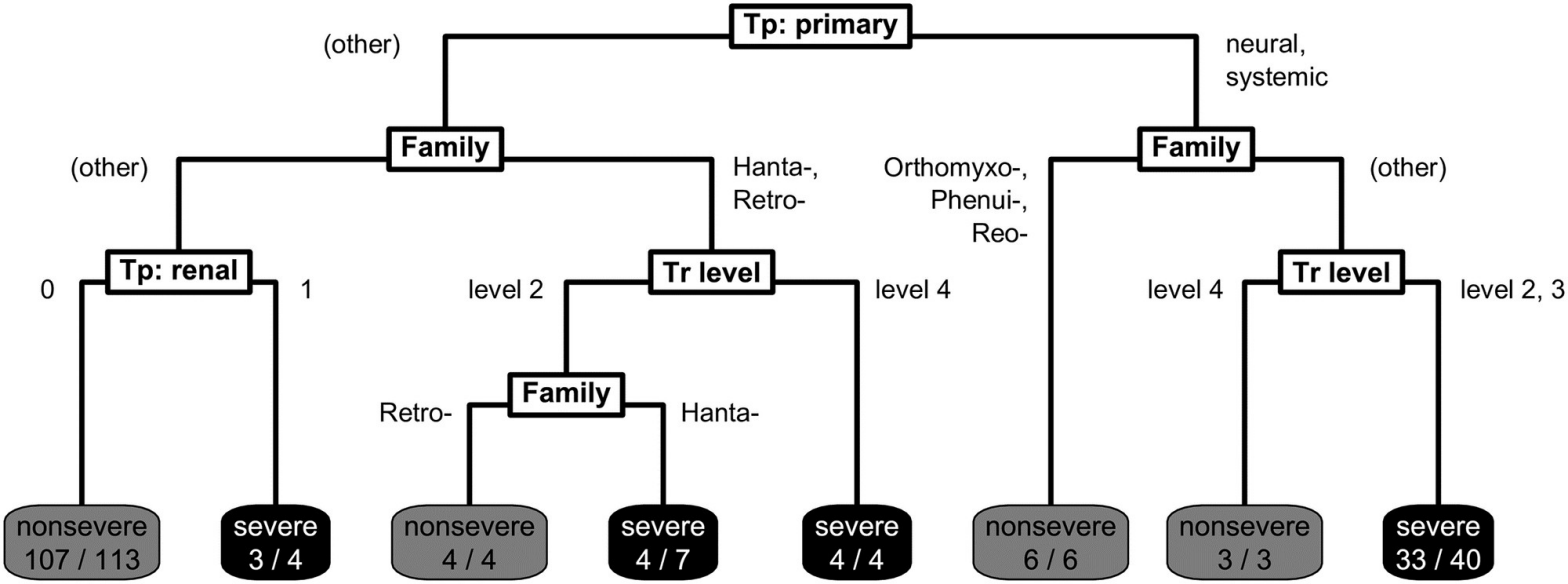
Final pruned classification tree predicting disease severity for 181 human RNA viruses. Final classification tree structure predicting virulence. Viruses begin at the top and are classified according to split criteria (white boxes) until reaching terminal nodes with the model’s prediction of disease severity, and the fraction of viruses following that path correctly classified is shown based on literature-assigned ratings (shaded boxes). ‘Tp: primary’ denotes primary tissue tropism, ‘Tr level’ denotes level of human-to-human transmissibility, and ‘Tp: renal’ denotes having a known renal tissue tropism. Tp, tropism; Tr, transmissibility.

To find predictive risk factors for virulence, we first divided the 212 virus species into a single training (*n* = 181) and test set (*n* = 31) partition based on taxonomy and severity to minimise potential biases from trait imbalances between sets. Using this training set, we then constructed a single classification tree that aimed to optimally classify viruses in virulence based on their ecological traits. The final pruned classification tree included variables relating to transmissibility, tissue tropism, and taxonomy (Fig 2). Severe disease was predicted by the model for four generalised groups: i) viruses with a neural or systemic primary tropism with limited human-to-human transmissibility (excluding orthomyxoviruses, phenuiviruses, and reoviruses); ii) viruses known to have a renal tropism (primary or otherwise); iii) hantaviruses; and iv) retroviruses with sustained human-to-human transmissibility.

### Random forest risk factor analysis

Although the illustrated classification tree identified several risk factors, this represents one of many possible trees because tree structure is dependent on the exact sampling partition between training and test data. We therefore constructed a random forest model containing 5,000 individual trees, each built using a bootstrapped sample of the training data and a randomly restricted subset of predictors, and repeated this approach over 200 alternative training/test set partitions.

**Fig 3:**
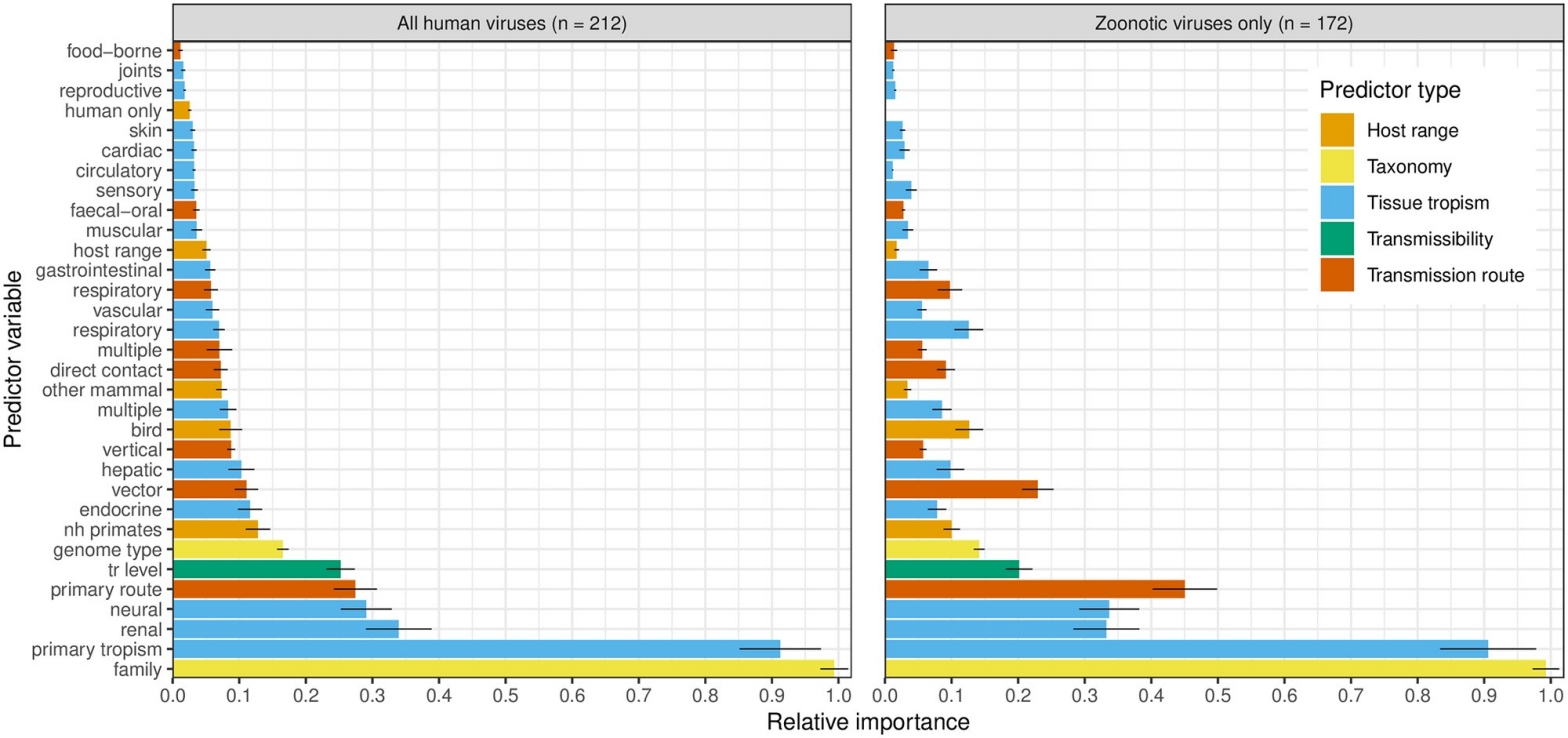
Variable importance from random forest models. Importance of each variable in predicting virulence in random forest models applied to all known human RNA viruses and zoonotic viruses only, calculated as the average decrease in Gini impurity following a tree split based on that predictor and scaled against the most informative predictor within each random forest to give a relative measure. Points denote mean values across 200 random forest models with alternative training/test partitions. Error bars denote ± 1 standard deviation. Colour key denotes type of predictor variable. Supporting data are available via figshare: 10.6084/m9.figshare.7406441.v3 (https://figshare.com/articles/Data_and_supporting_R_script_for_Tissue_Tropism_and_Transmission_Ecology_Predict_Virulence_of_Human_RNA_Viruses/7406441/3). nh, nonhuman; tr, transmissibility.

Averaging over these bootstrapped random forests, the most informative predictor variables for classifying virulence were taxonomic family and primary tissue tropism (Fig 3). However, primary transmission route, human-to-human transmissibility level, and having a known neural or renal tropism were also relatively informative, broadly mirroring the risk factors observed in the single tree. Host range predictors were generally uninformative. To identify whether virulence risk factors might differ for non-human–adapted viruses, we repeated our machine learning analysis for only those viruses with known or suspected zoonotic transmission. For zoonotic viruses, the most informative predictors were similar (Fig 3), though transmission route variables (primary transmission route, having known vector-borne transmission) appeared to increase in relative importance.

**Fig 4:**
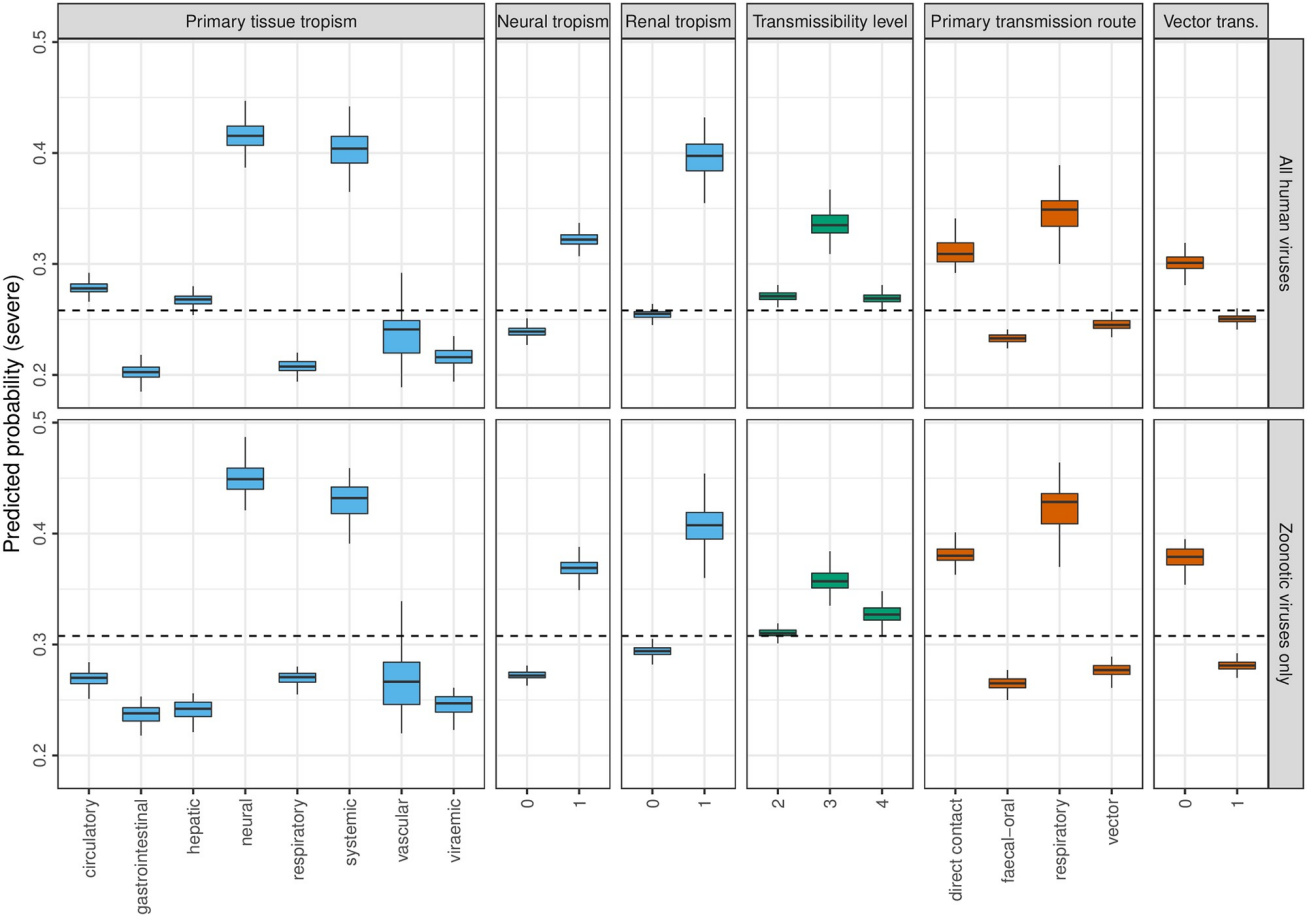
Partial dependence from random forest models in predicting severe virulence. Predicted probability of classifying virulence as ‘severe’ for each of the most informative risk factors in random forest models applied to all known human RNA viruses and zoonotic viruses only (primary tissue tropism, any known neural tropism, any known renal tropism, level of human-to-human transmissibility, primary transmission route, and any known vector-borne transmission). Predicted probabilities are marginal, i.e., averaging over any effects of other predictors. Boxes denote distribution of probabilities across 200 random forest models with alternative training/test partitions, with heavy lines denoting median probability. Dashed line denotes raw prevalence of ‘severe’ virulence rating among the respective training datasets. Colour key denotes predictor variable type as in Fig 3, i.e., blue = tissue tropism, green = transmissibility, red = transmission route. Supporting data are available via figshare: 10.6084/m9.figshare.7406441.v3 (https://figshare.com/articles/Data_and_supporting_R_script_for_Tissue_Tropism_and_Transmission_Ecology_Predict_Virulence_of_Human_RNA_Viruses/7406441/3).

To quantify the effects of the most informative risk factors, averaged partial dependence was extracted from the random forests, describing the marginal predicted probabilities of severe virulence associated with each virus trait (Fig 4, S2 Table). Averaging across other predictors, viruses having tissue tropisms within neural or renal systems or systemic across multiple organ systems presented the highest risk of severe virulence, whilst respiratory and gastrointestinal tropisms presented the lowest risk. An increased probability of severe virulence was also observed for viruses transmitted by direct contact or respiratory routes and those with known but limited human-to-human transmissibility. When restricted to zoonotic viruses, patterns of partial dependence were mostly similar to those observed for all human viruses (Fig 4).

### Model performance in predicting viral virulence

**Fig 5:**
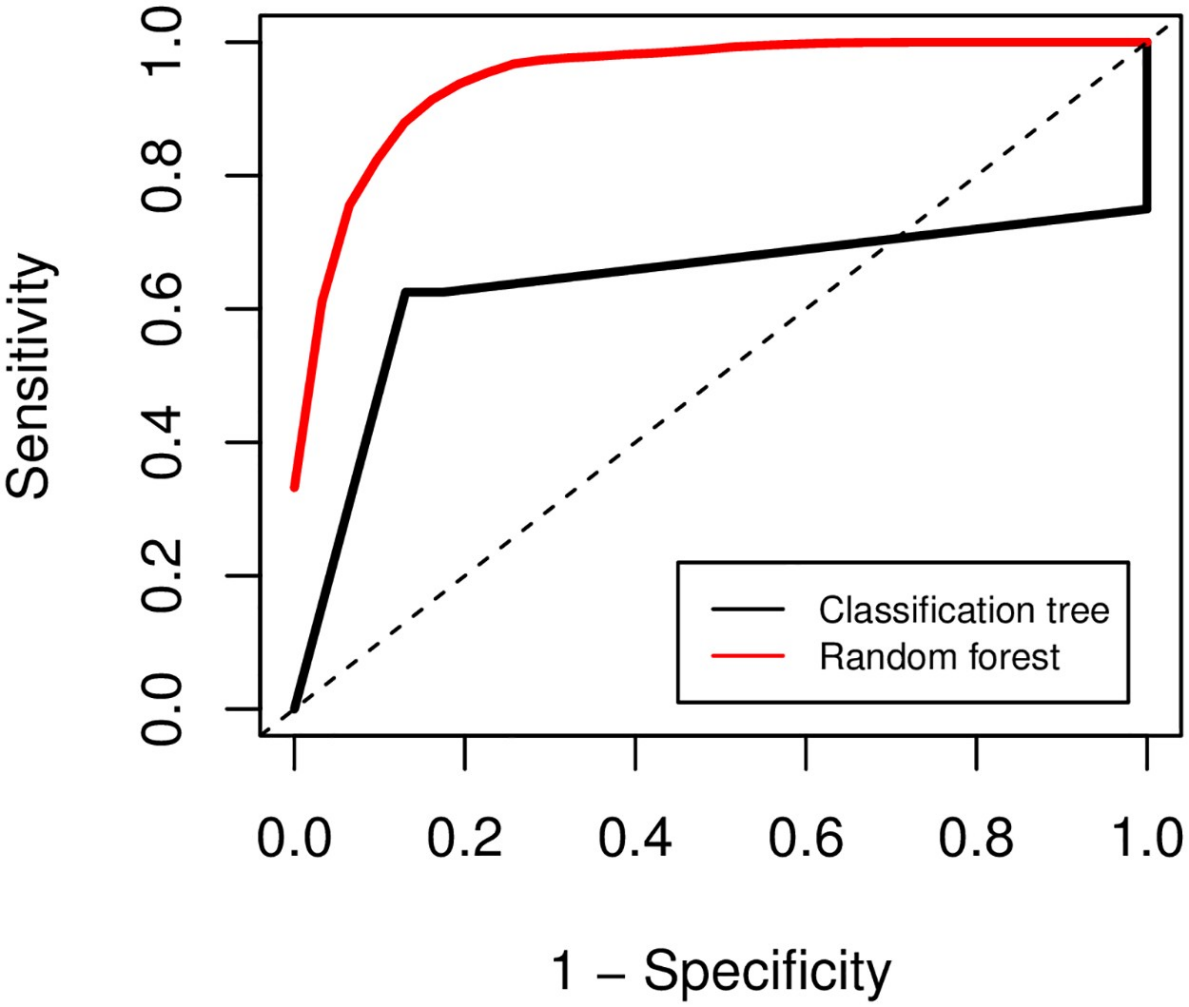
Receiver operating characteristic curve for tree-based machine learning models. Plotted models in predicting virulence in test set(s) for the single classification tree (bold black line) and averaged random forest models (bold red line) over 200 training/test set partitions. *y* Axis denotes sensitivity (or true positive rate; proportion of viruses rated ‘severe’ by literature protocol that were correctly predicted as ‘severe’ by the model), and *x* axis denotes 1 –specificity (or false positive rate; proportion of viruses rated ‘nonsevere’ by literature protocol that were incorrectly predicted as ‘severe’ by the model). Dashed black line indicates null expectation (i.e., a model with no discriminatory power). Model profiles further toward the top left indicate a better predictive performance.

Although the single classification tree model predicted its training set well, it did not appear generalisable to novel data within its test set. The single tree correctly predicted virulence ratings from literature-based criteria for 24 of 31 viruses in its test set, giving a resulting accuracy of 77.4% (95% confidence interval [CI]: 58.9%–90.4%), no evident improvement on the null model assigning all viruses as nonsevere (null accuracy = 74.2%). The random forest approach gave better predictive performance, correctly predicting virulence with a mean accuracy of 89.4% across all training/test partitions (95% CI: 72.0%–97.0%), significantly greater than the null accuracy (one-tailed one-sample proportion test, *p* = 0.041). The random forest approach also achieved superior performance when considering sensitivity, specificity, true skill statistic, and the negative predictive value as a performance measure prioritising correct classification of ‘severe’-rated viruses (Table 1). The random forests also outperformed the classification tree in area under the receiver operating characteristic curve (AUROC) (Table 1, Fig 5).

**Table 1:** Predictive performance metrics for classification tree and random forest model. Sensitivity, specificity, NPV (proportion of ‘nonsevere’ predictions that correctly matched literature rating), TSS (sensitivity + specificity − 1), and AUROC for predictive model methods applied to predict virulence of viruses within the test set. Random forest diagnostics indicate mean values across 200 training/test partitions. Supporting data are available via figshare: 10.6084/m9.figshare.7406441.v3 (https://figshare.com/articles/Data_and_supporting_R_script_for_Tissue_Tropism_and_Transmission_Ecology_Predict_Virulence_of_Human_RNA_Viruses/7406441/3).

Model	Sensitivity	Specificity	NPV	TSS	AUROC
Classification tree	0.625	0.826	0.864	0.451	0.636
Random forest	0.776	0.935	0.924	0.712	0.955

**Abbreviations**: AUROC, area under the receiver operating characteristic curve; NPV, negative predictive value; TSS, true skill statistic.

Nineteen of 139 viruses featured in test set partitions were misclassified from averaged random forest predictions (S1 Table): seven viruses rated as severe from literature protocols that were predicted to be nonsevere and 12 nonsevere viruses predicted to be severe. Misclassifications from the random forest occurred most frequently within the flaviviruses and orthohantaviruses (S1 Table), though misclassifications did not appear to occur disproportionately between genera (Fisher’s exact, 1,000 simulations, *p* = 0.930).

The observed predictor importance and risk factor directions were robust to constructing random forest models for subsets of viruses, removing those with low-certainty data or data from serological evidence only (S1 and S2 Figs), and similar performance diagnostics were obtained (S3 Table), though transmission route predictors appeared less informative when considering only viruses with at least 20 known cases. Redefining our virulence measure to integrate information on known fatalities and differences with subspecies or strains in an ordinal ranking system (S4 Table) did not improve predictive performance (S5 Table). Using alternative virulence measurements, the most informative variables and virus traits predicting severity showed good agreement with those of the main analysis (S3 and S4 Figs).

## Discussion

We present the first comparative analysis of virulence across all known human RNA virus species to our knowledge. We find that disease severity is nonrandomly distributed across virus families and that beyond taxonomy, severe disease is predicted by risk factors of tissue tropism and, to a lesser extent, transmission route and level of human-to-human transmissibility. In both classification tree and random forest models, viruses were more likely to be predicted to cause severe disease if they caused systemic infections, had neural or renal tropism, transmitted via direct contact or respiratory routes, or had limited capability to transmit between humans (0 < R_0_ ≤ 1). These risk factors were robust to alternative modelling methods, alternative definitions of virulence, and exclusions of poor-quality data.

### Ecology and evolution of risk factor traits

Primary tissue tropism was the most informative nontaxonomic risk factor (Fig 3) and the first split criteria in the classification tree (Fig 2), with specific neural tropism and generalised systemic tropism predicting severe disease (Fig 4). Few studies have directly predicted how tissue tropism should influence virulence. The identified risk factor tropisms could be explainable as a simple function of pathology occurring in sensitive or multiple tissues, respectively, increasing intensity of clinical disease. However, it has been suggested that an excessive, nonadapted virulence may result if infections occur within nontarget tissues that do not contribute to transmission [30], although the evolutionary determinants of tissue tropism are not well-understood [31]. Tissue tropism should be a key consideration for future comparative and evolutionary modelling efforts.

We also found viruses primarily transmitted by direct contact and respiratory routes to have a higher predicted probability of severe virulence than viruses transmitted by vector-borne or faecal–oral routes. Contrastingly, previous comparative analyses pooling several microparasite types, including a limited range of viruses, have shown positive associations between virulence and vector-borne transmission [17] or environmental survivability [18]. Ewald [17] suggested virulence has fewer costs to pathogen fitness if transmission can occur independent of host health and mobility, e.g., through arthropod vectors or contaminated water, though we did not observe support for this hypothesis in our analysis.

The relationship between virulence and transmissibility appears more complex. Firstly, random forest models suggested a lower risk of severe virulence for viruses with sustained human-to-human transmissibility (level 4) than self-limited transmissibility (level 3) (Fig 4). This appears consistent with hypothesised virulence–transmissibility tradeoffs [21,32,33] and suggests that the adaptation necessary to develop efficient human-to-human transmissibility could result in attenuation of virulence in RNA viruses. Sustained transmissibility appeared to positively predict severe disease for a specific subset of four viruses in the single classification tree (Fig 2), all retroviruses causing chronic syndromes (HIV 1 and 2 and primate T-lymphotropic virus 1 and 2), which are likely subject to different evolutionary dynamics—if disease occurs after the infectious period, virulence brings fewer costs to pathogens from host mortality, essentially ‘decoupling’ from transmission [23]. We note only three nonchronic level 4 viruses rated severe: severe acute respiratory syndrome-related coronavirus, yellow fever virus, and Zaire ebolavirus.

Although cross-species infections incapable of onward transmission (sometimes termed ‘dead-end’ infections) can result in high virulence because without coevolution, viral phenotypes within the novel host will be nonadapted—i.e., a ‘coincidental’ by-product [23,24]—we did not observe viruses incapable of human-to-human transmission (level 2) to be comparatively more virulent. This may suggest that if virulence is entirely unselected in dead-end infections, phenotypic levels of virulence could just as easily turn out to be ‘coincidentally’ low.

Taxonomic family being a highly informative predictor in the random forests implies that there is a broad phylogenetic signal to virulence, but it is also highly likely that the explanatory power represents a proxy for many other phylogenetically conserved viral traits that are challenging to implement in comparative analyses of this scale, such as variation at the proteomic, transcriptomic, or genomic level or further data beyond simple categorisations, e.g., specific arthropod vector species. Untangling these sources of variation from different scales of traits will be a critical next step in predictive modelling of viral virulence.

### Analytical limitations

We acknowledge several limitations to the quality of our data, as with any broad comparative analysis. Risk factor data were problematic or missing for certain viruses, e.g., natural transmission route for viruses only known to infect humans by accidental occupational exposure and tissue tropism for viruses only known from serological evidence. However, the consistency of findings between alternative, stricter definitions of virulence and data subsets removing viruses with suspected data quality issues suggests scarcity of data does not bias our analyses.

Virulence also exhibits substantial variation at the subspecies level, i.e., between strains or variants. For example, severity of Lassa virus disease superficially varies with infection route and geography, though this appears to be driven by variation between genotypes [34]. Confirmatory analyses at a finer resolution would validate our identified risk factors, e.g., phylogenetic trait models of individual genera or species. Furthermore, clinical symptoms are also subject to traits of the host individual, e.g., immunocompetence, age, and microbiome [35,36]. Our risk factor analysis brings a novel, to our knowledge, top-down perspective on virulence at the broadest level, though caution must be exerted in extrapolating the risk factors we find to dynamics of specific infections.

### Implications for public health

The value of predictive modelling as an inexpensive and rapid tool for risk assessments during early emergence is increasingly recognised [16]. Instances in which machine learning model predictions do not match outcomes could indicate likely candidates for outcome class changes, e.g., future reservoir hosts for zoonotic disease [37], and we note severe virulence was predicted for 12 viruses rated ‘nonsevere’ from literature protocols (S1 Table).

However, our models have restricted function in predicting the virulence of a newly identified virus, particularly if human infections are not yet recognised. Taxonomy may be easily accessible and applicable to give simple virulence estimates. However, the most informative nontaxonomic predictors, tissue tropism and transmission route, are not likely to be identified with confidence before clinical observations of virulence. One way to address this information gap would be use of available data from animal infections, assuming that tissue tropism and transmission route do not differ between human and nonhuman hosts. Alternatively, predictor data might be imputed from the nearest-related known virus, particularly for traits that appear highly phylogenetically conserved such as tissue tropism [31].

A more powerful future approach lies in the potential predictability of tissue tropism based on cell receptors and, more challengingly, of cell receptors based on viral proteomics or sequence data [38], an increasingly accessible information source during early emergence following advances in genomic sequencing methods [39]. The exact links between tissue tropism, cell receptors, and nucleotide sequences are currently a critical knowledge gap and a potentially informative focus for future predictive efforts. A further key area requiring development is the possibility of inferring virulence directly from aspects of sequence data, e.g., genome composition biases, which have recently demonstrated the potential to predict reservoir host taxa and arthropod vectors via machine learning [40].

More widely, our analysis brings a novel, to our knowledge, focus that complements comparative models predicting other aspects of the emergence process such as zoonotic transmission [8,9,37,41], propagation within humans [10,11], or geographic hotspots [42,43]. After continued calls for model-informed strategy, predictive studies are now beginning to shape surveillance and prevention with respect to emerging zoonoses [16,44], with virulence being been suggested as a factor to direct viral surveillance [45], albeit in nonhuman hosts. The virulence risk factors we identify suggest that broadly targeting direct contact or respiratory transmission interfaces within ecological systems and/or tailoring detection assays towards certain virus families (e.g., Hantaviridae) or tissues (e.g., neural tissue) could contribute to a viable strategy to detect future virulent zoonoses.

### Conclusion

This work adds to the comparative and predictive modelling efforts surrounding emerging infectious diseases. Here, we contribute a novel, to our knowledge, focus on ecological predictors of virulence of human RNA viruses, which can be combined in holistic frameworks with other models such as those predicting emergence dynamics. As a predictive model, the featured random forests offer valuable inference into the evolutionary determinants of virulence in newly emerging infections. We propose that future predictive studies and preparedness initiatives with respect to emerging diseases should carefully consider potential for human virulence.

## Materials and methods

### Data collection

For each of the 214 recognised human-infective RNA virus species, following standardised data compilation efforts and critical assessment protocols [5], data on virulence and potential risk factors were collected via a systematic search and review of clinical and epidemiological literature. The following were consulted in turn: clinical virology textbooks [46–48]; references from the data set described by [5]; and literature searches using Google Scholar (search terms: 1) [virus name] AND human, 2) [virus name] AND human AND case, 3) [virus name] AND human AND [fatal* OR death], 4) [virus name] AND human AND [tropi* or isolat*]). Searches 3 and 4 were carried out only when fatality or tropism data, respectively, were not already found from previous sources. Data collection and virus name search terms included the full species name, any synonyms or subspecies (excluding vaccine strains), and the standard virus abbreviation as given by ICTV Online Virus Taxonomy [49].

Although many possible measurements of virulence have been proposed [50,51], even simple metrics like CFR have not been calculated for the majority of human RNA virus species. Therefore, virulence was rated using a simple two-category measure of severity of typical disease in humans. We rated viruses as ‘severe’ if they firstly had ≥5% CFR when data were available (159/214 viruses, including those with zero CFR); otherwise, we rated viruses as ‘severe’ if they had frequent reports of hospitalisation, were associated with significant morbidity from certain conditions (haemorrhagic fever, seizures/coma, cirrhosis, AIDS, hantavirus pulmonary syndrome, HTLV-associated myelopathy), or were explicitly described as ‘severe’ or ‘causing severe disease’ (S1 Table). We rated viruses as ‘nonsevere’ if none of these conditions were met. We note that this led to ‘nonsevere’ ratings for some viruses with clinically severe but rare syndromes; e.g., dengue virus can cause haemorrhagic dengue fever, though this is much rarer than typical acute dengue fever [46,47]. To address this, data were also collected on whether the virus has caused fatalities in vulnerable individuals (defined as age 16 and below or 60 and above, immunosuppressed, having comorbidities, or otherwise cited as being ‘at-risk’ by sources for specific viruses) and in healthy adults and whether any ‘nonsevere’ virus has atypically severe strains (e.g., most infections with viruses within the species *Human enterovirus C* cause mild disease; however, poliovirus, which causes severe paralytic disease, is also classified under this species). These were examined both individually and within a composite six-rank system (S4 Table).

Data were compiled for four main risk factors: transmission route(s) and tissue tropism(s), sourced from literature search exercises as described, and extent of human-to-human transmissibility and host range, sourced directly from [5]. Although previous studies also predict virulence to vary with other traits, e.g., environmental survivability [18], paucity of data or nestedness within taxonomic family prevented their inclusion in our analysis. Firstly, primary transmission route was categorised as the dominant route the virus is transmitted by: vector-borne (excluding mechanical transmission), direct contact, faecal–oral, or respiratory transmission. Primary tissue tropism was similarly categorised as the dominant organ system the virus typically infects or targets, specified as neural, gastrointestinal, hepatic, respiratory, circulatory, vascular, or ‘systemic’ (typical infection within multiple organ systems with no clear, single dominant tropism). However, many human viruses are known from isolation from blood or serum, with no further evidence of specific tissue tropisms (*n* = 69). Therefore, we also included an additional ‘viraemia’ category in the primary tissue tropism predictor to indicate only blood presence was known.

Secondly, binary variables were also constructed, denoting whether viruses had ever been observed to utilise a) multiple transmission routes/tissue tropisms and b) each individual transmission route and tropism, including additional categories that were never among the primary routes/tropisms (food-borne and vertical transmission; renal, cardiac, joint, reproductive, sensory, skin, muscular, and endocrine tropism). We accepted isolation of the virus, viral proteins or genetic material, or diagnostic symptoms of the virus (such as characteristic histological damage) as evidence of infection within an organ system but did not accept generalised symptoms such as inflammation.

Human-to-human transmissibility was specified using infectivity/transmissibility levels, based on previous conceptual models and a systematic compilation and review of evidence [4,5,12]. Level 2 denotes a virus capable of infecting humans but not transmitting between humans (R_0_ = 0), level 3 denotes a virus with limited human-to-human transmissibility (0 < R_0_ ≤ 1), and level 4 denotes a virus with sustained human-to-human transmissibility (R_0_ ≥ 1). Host range was specified as either ‘narrow’ (infection known only within humans or humans plus nonhuman primates) or ‘broad’ (infection known in mammals or animals beyond primates) [5]. Binary variables were also sourced as to whether infection was known within a) humans only, b) nonhuman primates, c) other mammals, and d) birds.

To identify potential differences in risk factors between adapted and nonadapted viruses, we also categorised whether each virus was zoonotic. We considered a virus to be zoonotic if it had transmissibility level 2 or 3 or had transmissibility level 4 and was known to infect nonhuman hosts (excluding anthroponotic viruses, e.g., measles morbillivirus). We also conservatively considered viruses to be zoonotic if zoonotic potential was suspected but data-deficient, e.g., rotavirus A–C. All virulence and risk factor data pertained to natural or unintentional artificially acquired human infection only, and data from intentional human infection, animal infection, and in vitro infection were not considered. Viral taxonomy was included in analyses by specifying both genome type and taxonomic family as predictors. All virulence and risk factor data are available via figshare: 10.6084/m9.figshare.7406441.v3 (https://figshare.com/articles/Data_and_supporting_R_script_for_Tissue_Tropism_and_Transmission_Ecology_Predict_Virulence_of_Human_RNA_Viruses/7406441/3).

### Machine learning risk factor analysis

Firstly, the 212 retained virus species were split into a training set for model fitting and a test set for model evaluation. In order to avoid bias from an imbalance between types of viruses assigned to training and test sets, our selection was based on random sampling, stratified by genus–severity rating combinations. We sampled at a ratio of 75:25, i.e., for the four known severe viruses in the genus *Ebolavirus*, three were randomly assigned to the training set and the remaining one assigned to the test set. If a genus–severity combination contained less than four viruses, all defaulted to the test set. Comparative risk factor analyses were firstly carried out by constructing a classification tree using the R package ‘rpart’ v4.1–11 [52]. Classification trees are a simple form of machine learning models that aim to optimally classify data points into their correct category of outcome variable based on a structure of binary predictor splits. Tree-based methods are well-suited for comparative analyses in which confounding often results from taxonomic signal or suites of otherwise co-occurring traits because their high structure can intuitively fit complex nonlinear interactions and local effects.

A tree model was fitted to the training set to predict virulence ratings by ‘recursive partitioning’, the repeated splitting of the data set using every possible binary permutation of each predictor, and retaining the split that minimises the Gini impurity [53], defined as $1-\sum_{i=1}^{n}{p(x_{i})}^{2}$ for outcome variable *x* with *n* possible ratings and *p*(*x*_*i*_) denoting proportion of data with rating *i*, which is equal to zero for perfectly separated data. To prevent overfitting, the tree was pruned back to the optimal branching size, taken as the most common consensus size over 1,000 repeats of 10-fold cross-validation. To validate the predictive power of the classification tree, predictions of virulence rating were generated when applied to the test set. Tree accuracy was then calculated, comparing the proportion of correct predictions compared to literature-assigned ratings (assuming these to be 100% accurate as the ‘gold standard’ or ‘ground truth’). Because virulence ratings were imbalanced (i.e., only a minority of viruses cause severe disease, so correct nonsevere classifications are likely to be achieved by chance), accuracy was directly compared to the null model, i.e., a model with no predictors that predicted ‘nonsevere’ for all viruses. Additional diagnostics of interest (sensitivity, specificity, negative predictive value, and true skill statistic [54]) were also obtained.

Although classification trees have the advantage of presenting an interpretable schematic of risk factor effects and directions, individual tree structures may be sensitive to particular data points and have no intuitive measures of uncertainty. We therefore generated a further 200 partitions of our data into alternative training/test sets using the random stratified sampling procedure described. Then, for each partition, we constructed a random forest, an ensemble collection of a large number of bootstrapped classification trees [55]. Having many predictor variables compared to the relatively limited and fixed number of human-infective RNA virus species, random forests handle such ‘large *p*, small *n*’ data architecture much more easily than traditional regression frameworks [56]. Missing data in all predictors were imputed using the R package ‘missForest’ v1.4 [57]. Using the R package ‘randomForest’ v4.6–12 [57], random forests were created containing 5,000 individual trees, each built using a bootstrapped sample of training data and restricted to a randomly selected subset of predictors (k = 5) at each branching split. The predictive power of the random forest approach was evaluated by averaging over the test set predictions from all partitions. Receiver operating characteristic curves were visualised and area under curves calculated to directly compare to the classification tree methodology.

Because of their high structuring, random forest models cannot give a simple parametric predictor effect size and direction (e.g., an odds ratio). Instead, potential virulence risk factors were evaluated using two metrics: variable importance and partial dependence. Variable importance is calculated as the mean decrease in Gini impurity following tree splits on the predictor and can be considered as how informative the risk factor was towards correctly predicting virulence. Partial dependence is calculated as the mean relative change in log-odds of predicting severe virulence, which were converted to predicted probabilities of severity associated with each risk factor. Partial dependence describes marginal effects averaging across any influence of other predictors, and, as such, point estimates may not reflect any complex risk factor interactions. Therefore, to test hypotheses regarding virulence risk factors, we present both averaged random forest partial dependence and the less robust but more accessible single classification tree for its ease of interpretation in risk factor structure and directly compare the statistical validity of both methods by plotting receiver operating characteristic curves. All modelling was carried out in R v3.4.3 [58] with a supporting R script available via figshare: 10.6084/m9.figshare.7406441.v3 (https://figshare.com/articles/Data_and_supporting_R_script_for_Tissue_Tropism_and_Transmission_Ecology_Predict_Virulence_of_Human_RNA_Viruses/7406441/3).

## Supporting information

S1 Table Virulence literature rating data for human RNA virus training data set. Virulence data for 212 human virus species ordered by genome type and taxonomy, including disease severity rating and supporting criteria for viruses rated ‘severe’, whether virus is known to have caused fatalities in vulnerable individuals and/or otherwise healthy adults, and whether virus is known to have ‘severe’ strains if species is rated ‘nonsevere’. Both disease severity rating/supporting criteria following the literature protocol given in the main text and mean predicted probability of severe disease from the random forest models are given. Bold type denotes when predictions do not match literature-based ratings. Dashes indicate predictions were not generated because fewer than four viruses were observed with this genus–severity combination and virus always defaulted to training set. AIDS, acquired immunodeficiency syndrome; CFR, case fatality ratio; HFRS, hantavirus haemorrhagic fever with renal syndrome; HPS, hantavirus pulmonary syndrome; HTLV, human T-lymphotropic virus.
(PDF)

S2 Table Partial dependence from random forest models for all predictor variables. Partial dependence given as mean marginal relative change in log-odds and mean predicted probability of classifying virulence as ‘severe’ for all predictor variables from random forest models featuring all viruses and models featuring zoonotic viruses only.
(PDF)

S3 Table Diagnostics of random forest models using stringent data subsets. Predictive performance metrics of random forest models applied to data subsets, excluding viruses with low-certainty data (*n* denotes number of viruses excluded). Diagnostics indicate mean values across 200 training/test partitions sampled separately for each data subset. Otherwise, random forest methodology follows that of Materials and Methods. Supporting data are available via figshare: 10.6084/m9.figshare.7406441.v3 (https://figshare.com/articles/Data_and_supporting_R_script_for_Tissue_Tropism_and_Transmission_Ecology_Predict_Virulence_of_Human_RNA_Viruses/7406441/3).
(CSV)

S4 Table Six-rank system of classifying virulence for human RNA viruses. Six-rank system of classifying human RNA virus virulence with available data (specifically, severity rating from main text, fatalities in vulnerable individuals and healthy adults, and severe strains), along with example viruses and number of viruses fitting each exclusive rank’s criteria.
(PDF)

S5 Table Diagnostics of random forest models predicting alternative metrics of virulence. Predictive performance metrics of random forest models predicting alternative virulence measures using different two-category definitions of ‘severe’ (*n* denotes number of viruses considered ‘severe’ using that definition). Vulnerable individuals are defined as those age 16 and below, age 60 and above, immunosuppressed, having comorbidities, or otherwise cited as being ‘at-risk’. Ranks follow those given in Table S5. Diagnostics indicate mean values across 200 training/test partitions sampled separately for each virulence metric. Otherwise, random forest methodology follows that of Materials and Methods. Supporting data are available via figshare: 10.6084/m9.figshare.7406441.v3 (https://figshare.com/articles/Data_and_supporting_R_script_for_Tissue_Tropism_and_Transmission_Ecology_Predict_Virulence_of_Human_RNA_Viruses/7406441/3).
(CSV)

S1 Fig Variable importance from random forest models using stringent data subsets. Variable importance for virulence risk factors from random forest models applied to data sets, excluding a) viruses only known to infect humans from serological evidence (*n* = 36), b) viruses with <20 recognised human infections (*n* = 55), and c) viruses with poor data quality in at least one predictor (*n* = 71). Variable importance is calculated as the relative mean decrease in Gini impurity scaled against the most informative predictor within each model alongside importance from the main analysis for comparison. Points denote mean values across 200 training/test partitions. Error bars denote ± 1 standard deviation. Colour key denotes type of predictor variable. Supporting data are available via figshare: 10.6084/m9.figshare.7406441.v3 (https://figshare.com/articles/Data_and_supporting_R_script_for_Tissue_Tropism_and_Transmission_Ecology_Predict_Virulence_of_Human_RNA_Viruses/7406441/3).
(TIF)

S2 Fig Partial dependence from random forest models using stringent data subsets. Predicted probability of classifying virulence as ‘severe’ for each of the most informative risk factors from random forest models applied to data sets excluding a) viruses only known to infect humans from serological evidence (*n* = 36), b) viruses with <20 recognised human infections (*n* = 55), and c) viruses with poor data quality in at least one predictor (*n* = 71) alongside predicted probabilities from the main analysis for comparison. Probabilities given are marginal, i.e., averaging over any effects of other predictors. Because each data subset required resampling of the training and test partitions, note that raw prevalence of ‘severe’ virulence differed between each model (see S3 Table). Boxes denote distribution of probabilities across 200 training/test partitions, with heavy lines denoting median probability. Colour key denotes predictor variable type as in Fig 3, i.e., blue = tissue tropism, green = transmissibility, red = transmission route. Supporting data are available via figshare: 10.6084/m9.figshare.7406441.v3 (https://figshare.com/articles/Data_and_supporting_R_script_for_Tissue_Tropism_and_Transmission_Ecology_Predict_Virulence_of_Human_RNA_Viruses/7406441/3).
(TIF)

S3 Fig Variable importance from random forest models predicting alternative metrics of virulence. Variable importance for virulence risk factors from random forest models predicting alternative virulence measures using different two-category definitions of ‘severe’, calculated as the relative mean decrease in Gini impurity scaled against the most informative predictor within each model alongside importance from the main analysis for comparison. Points denote mean values across 200 training/test partitions. Error bars denote ± 1 standard deviation. Colour key denotes type of predictor variable. Supporting data are available via figshare: 10.6084/m9.figshare.7406441.v3 (https://figshare.com/articles/Data_and_supporting_R_script_for_Tissue_Tropism_and_Transmission_Ecology_Predict_Virulence_of_Human_RNA_Viruses/7406441/3).
(TIF)

S4 Fig Partial dependence from random forest models using predicting alternative metrics of virulence. Predicted probability of classifying virulence as ‘severe’ in alternative virulence measures for each of the most informative risk factors from random forest models alongside predicted probabilities from the main analysis for comparison. Probabilities given are marginal, i.e., averaging over any effects of other predictors. Because each measurement used a different two-category definition of ‘severe’, note that the raw prevalence of ‘severe’ virulence differed between each model (see S5 Table). Boxes denote distribution of probabilities across 200 training/test partitions, with heavy lines denoting median probability. Colour key denotes predictor variable type as in Fig 3, i.e., blue = tissue tropism, green = transmissibility, red = transmission route. Supporting data are available via figshare: 10.6084/m9.figshare.7406441.v3 (https://figshare.com/articles/Data_and_supporting_R_script_for_Tissue_Tropism_and_Transmission_Ecology_Predict_Virulence_of_Human_RNA_Viruses/7406441/3).
(TIF)

## Abbreviations

AUROC: area under the receiver operating characteristic curve
CFR: case fatality ratio
CI: confidence interval
ICTV: International Committee on Taxonomy of Viruses
MERS: Middle East respiratory syndrome
