# Supplementary material for: Tissue tropism and transmission ecology predict virulence of human RNA viruses
Source: PLoS Biol. 2019 Nov 26;17(11):e3000206. doi: 10.1371/journal.pbio.3000206 (PMC6879112; doi:10.1371/journal.pbio.3000206)
Supplement: S1 Table — Virulence data for 212 human virus species ordered by genome type and taxonomy, including disease severity rating and supporting criteria for viruses rated ‘severe’, whether virus is known to have caused fatalities in vulnerable individuals and/or otherwise healthy adults, and whether virus is known to have ‘severe’ strains if species is rated ‘nonsevere’. Both disease severity rating/supporting criteria following the literature protocol given in the main text and mean predicted probability of severe disease from the random forest models are given. Bold type denotes when predictions do not match literature-based ratings. Dashes indicate predictions were not generated because fewer than four viruses were observed with this genus–severity combination and virus always defaulted to training set. AIDS, acquired immunodeficiency syndrome; CFR, case fatality ratio; HFRS, hantavirus haemorrhagic fever with renal syndrome; HPS, hantavirus pulmonary syndrome; HTLV, human T-lymphotropic virus. (PDF) [file pbio.3000206.s001.pdf]

Family	Genus	Species	Severity rating (literature protocol)	Severity notes	Fatalities (vulnerable)	Fatalities (healthy adults)	Severe strains	Mean p(severe)	References
-ssRNA viruses									
Arenaviridae	*Mammarenavirus*	*Chapare mammarenavirus*	severe	Haemorrhagic fever, CFR 100% (1 case)	1	1	N/A	0.824	[1–3]
Arenaviridae	*Mammarenavirus*	*Guanarito mammarenavirus*	severe	Haemorrhagic fever, CFR 25%	1	1	N/A	0.849	[1,4]
Arenaviridae	*Mammarenavirus*	*Junín mammarenavirus*	severe	Haemorrhagic fever, CFR 15-30%	1	1	N/A	0.852	[1,4]
Arenaviridae	*Mammarenavirus*	*Lassa mammarenavirus*	severe	Haemorrhagic fever, CFR 15%	1	1	N/A	0.777	[4]
Arenaviridae	*Mammarenavirus*	*Lujo mammarenavirus*	severe	Haemorrhagic fever, CFR 80% (5 cases)	1	1	N/A	0.837	[5,6]
Arenaviridae	*Mammarenavirus*	*Lymphocytic choriomeningitis mammarenavirus*	nonsevere		1	1	0	—	[1,2,4]
Arenaviridae	*Mammarenavirus*	*Machupo mammarenavirus*	severe	Haemorrhagic fever, CFR 5-35%	1	1	N/A	0.841	[1,2,4]
Arenaviridae	*Mammarenavirus*	*Mobala mammarenavirus*	nonsevere		0	0	0	—	[7]
Arenaviridae	*Mammarenavirus*	*Pichindé mammarenavirus*	nonsevere		0	0	0	—	[4]
Arenaviridae	*Mammarenavirus*	*Sabiá mammarenavirus*	severe	Haemorrhagic fever, CFR 33% (3 cases)	1	1	N/A	0.844	[1,2,4]
Arenaviridae	*Mammarenavirus*	*Whitewater Arroyo mammarenavirus*	severe	Haemorrhagic fever, CFR 100% (3 cases)	1	1	N/A	0.855	[1,4,8]
Bornaviridae	*Bornavirus*	*Mammalian 1 bornavirus*	nonsevere		0	0	0	—	[4]
Bornaviridae	*Bornavirus*	*Mammalian 2 bornavirus*	severe	CFR 100% (3 cases)	1	0	N/A	—	[9]
Filoviridae	*Ebolavirus*	*Bundibugyo ebolavirus*	severe	Haemorrhagic fever, CFR 35%	1	1	N/A	0.858	[10,11]
Filoviridae	*Ebolavirus*	*Reston ebolavirus*	nonsevere		0	0	0	—	[2,4]
Filoviridae	*Ebolavirus*	*Sudan ebolavirus*	severe	Haemorrhagic fever, CFR 41-65%	1	1	N/A	0.879	[1,4]
Filoviridae	*Ebolavirus*	*Tai forest ebolavirus*	severe	Haemorrhagic fever	0	0	N/A	0.864	[1,4]

Filoviridae	*Ebolavirus*	*Zaire ebolavirus*	severe	Haemorrhagic fever, CFR 61-90%	1	1	N/A	0.827	[4]
Filoviridae	*Marburgvirus*	*Marburg marburgvirus*	severe	Haemorrhagic fever, CFR 23-90%	1	1	N/A	—	[4]
Hantaviridae	*Orthohantavirus*	*Andes orthohantavirus*	severe	HPS	1	1	N/A	0.865	[2]
Hantaviridae	*Orthohantavirus*	*Bayou orthohantavirus*	severe	HPS	1	0	N/A	0.860	[2,12]
Hantaviridae	*Orthohantavirus*	*Black creek canal orthohantavirus*	severe	HPS	0	0	N/A	0.859	[2]
Hantaviridae	*Orthohantavirus*	*Choclo orthohantavirus*	severe	HPS, CFR 10-25%	1	1	N/A	0.870	[13–15]
Hantaviridae	***Orthohantavirus***	***Dobrava-Belgrade orthohantavirus***	**severe**	HFRS, CFR 5-35%	1	1	N/A	**0.231**	[2,4]
Hantaviridae	***Orthohantavirus***	***Hantaan orthohantavirus***	**severe**	HFRS, CFR 5-15%	1	1	N/A	**0.172**	[4]
Hantaviridae	*Orthohantavirus*	*Laguna Negra orthohantavirus*	severe	HPS	1	1	N/A	0.862	[1,4,16]
Hantaviridae	*Orthohantavirus*	*Puumala orthohantavirus*	nonsevere		1	1	0	0.435	[2,4]
Hantaviridae	***Orthohantavirus***	***Sangassou orthohantavirus***	**severe**	HFRS	0	0	N/A	**0.330**	[17]
Hantaviridae	***Orthohantavirus***	***Seoul orthohantavirus***	**nonsevere**		1	1	0	**0.503**	[4]
Hantaviridae	*Orthohantavirus*	*Sin Nombre orthohantavirus*	severe	HPS, CFR 32-75%	1	1	N/A	0.877	[1,4]
Hantaviridae	*Orthohantavirus*	*Thailand orthohantavirus*	nonsevere		0	0	0	0.344	[18]
Hantaviridae	***Orthohantavirus***	***Thottapalayam orthohantavirus***	**severe**	Only known case required hospitalisation	0	0	N/A	**0.242**	[19]
Hantaviridae	*Orthohantavirus*	*Tula orthohantavirus*	nonsevere		0	0	0	0.316	[20]
Nairoviridae	*Orthonairovirus*	*Crimean-Congo haemorrhagic fever orthonairovirus*	severe	Haemorrhagic fever, CFR 30%	1	1	N/A	—	[1,4]
Nairoviridae	*Orthonairovirus*	*Dugbe orthonairovirus*	nonsevere		0	0	0	—	[2]
Nairoviridae	*Orthonairovirus*	*Thiafora orthonairovirus*	nonsevere		0	0	0	—	[21,22]

Orthomyxoviridae	*Influenzavirus A*	*Influenza A virus*	nonsevere		1	1	1	—	[1,4]
Orthomyxoviridae	*Influenzavirus B*	*Influenza B virus*	nonsevere		1	1	0	—	[4]
Orthomyxoviridae	*Influenzavirus C*	*Influenza C virus*	nonsevere		0	0	0	—	[4]
Orthomyxoviridae	*Thogotovirus*	*Dhori virus*	nonsevere		0	0	0	—	[23]
Orthomyxoviridae	*Thogotovirus*	*Thogoto virus*	nonsevere		1	0	0	—	[24]
Paramyxoviridae	*Avulavirus*	*Avian avulavirus 1*	nonsevere		1	0	0	—	[1,25]
Paramyxoviridae	*Henipavirus*	*Hendra henipavirus*	severe	CFR 57% (7 cases)	1	1	N/A	—	[4,26]
Paramyxoviridae	*Henipavirus*	*Nipah henipavirus*	severe	CFR 10-75%	1	1	N/A	—	[1,4]
Paramyxoviridae	*Morbillivirus*	*Canine morbillivirus*	nonsevere		0	0	0	—	[27]
Paramyxoviridae	*Morbillivirus*	*Measles morbillivirus*	nonsevere		1	1	0	—	[2,4]
Paramyxoviridae	*Respirovirus*	*Human respirovirus 1*	nonsevere		0	0	0	—	[4]
Paramyxoviridae	*Respirovirus*	*Human respirovirus 3*	nonsevere		1	0	0	—	[4]
Paramyxoviridae	*Rubulavirus*	*Human rubulavirus 2*	nonsevere		0	0	0	0.047	[4]
Paramyxoviridae	*Rubulavirus*	*Human rubulavirus 4*	nonsevere		0	0	0	0.046	[4]
Paramyxoviridae	*Rubulavirus*	*Mammalian rubulavirus 5*	nonsevere		0	0	0	0.050	[4,28]
Paramyxoviridae	***Rubulavirus***	***Mumps rubulavirus***	**nonsevere**		1	1	0	**0.777**	[4]
Paramyxoviridae	*Rubulavirus*	*Simian rubulavirus*	nonsevere		0	0	0	0.047	[29]
Paramyxoviridae	*Rubulavirus*	*Sosuga rubulavirus*	severe	Single known case required hospital admission	0	0	N/A	—	[30]
Paramyxoviridae	*Rubulavirus*	*Tioman rubulavirus*	nonsevere		0	0	0	0.044	[31]

Peribunyaviridae	*Orthobunyavirus*	*Bunyamwera orthobunyavirus*	nonsevere		0	0	0	0.048	[2,4]
Peribunyaviridae	*Orthobunyavirus*	*Bwamba orthobunyavirus*	nonsevere		0	0	0	0.045	[2]
Peribunyaviridae	*Orthobunyavirus*	*California encephalitis orthobunyavirus*	severe	High frequency of severe symptoms (seizures, coma)	1	1	N/A	—	[4]
Peribunyaviridae	*Orthobunyavirus*	*Caraparu orthobunyavirus*	nonsevere		0	0	0	0.050	[2]
Peribunyaviridae	*Orthobunyavirus*	*Catu orthobunyavirus*	nonsevere		0	0	0	0.047	[2]
Peribunyaviridae	*Orthobunyavirus*	*Guama orthobunyavirus*	nonsevere		0	0	0	0.045	[2]
Peribunyaviridae	*Orthobunyavirus*	*Guaroa orthobunyavirus*	nonsevere		0	0	0	0.047	[2]
Peribunyaviridae	*Orthobunyavirus*	*Kairi orthobunyavirus*	nonsevere		0	0	0	0.049	[32]
Peribunyaviridae	*Orthobunyavirus*	*Madrid orthobunyavirus*	nonsevere		0	0	0	0.047	[2,4,33]
Peribunyaviridae	*Orthobunyavirus*	*Marituba orthobunyavirus*	nonsevere		0	0	0	0.055	[2,34]
Peribunyaviridae	*Orthobunyavirus*	*Nyando orthobunyavirus*	nonsevere		0	0	0	0.044	[2,35]
Peribunyaviridae	*Orthobunyavirus*	*Oriboca orthobunyavirus*	nonsevere		0	0	0	0.042	[2,34]
Peribunyaviridae	*Orthobunyavirus*	*Oropouche orthobunyavirus*	nonsevere		0	0	0	0.045	[1,4]
Peribunyaviridae	*Orthobunyavirus*	*Patois orthobunyavirus*	nonsevere		0	0	0	0.047	[36]
Peribunyaviridae	*Orthobunyavirus*	*Shuni orthobunyavirus*	nonsevere		0	0	0	0.044	[2]
Peribunyaviridae	*Orthobunyavirus*	*Tacaiuma orthobunyavirus*	nonsevere		0	0	0	0.050	[2,37]
Peribunyaviridae	*Orthobunyavirus*	*Wyeomyia orthobunyavirus*	nonsevere		0	0	0	0.044	[2,38]
Phenuiviridae	*Phlebovirus*	*Candiru phlebovirus*	nonsevere		0	0	0	0.119	[2,39]
Phenuiviridae	*Phlebovirus*	*Punta Toro phlebovirus*	nonsevere		0	0	0	0.109	[1,2]

Phenuiviridae	***Phlebovirus***	***Rift Valley fever phlebovirus***	**nonsevere**		1	1	0	**0.829**	[2]
Phenuiviridae	*Phlebovirus*	*Sandfly fever Naples phlebovirus*	nonsevere		0	0	0	0.130	[1,2]
Phenuiviridae	*Phlebovirus*	*SFTS phlebovirus*	severe	CFR 15%	1	1	N/A	—	[40–43]
Phenuiviridae	*Phlebovirus*	*Uukuniemi phlebovirus*	nonsevere		0	0	0	0.205	[2]
Pneumoviridae	*Metapneumovirus*	*Avian metapneumovirus*	nonsevere		0	0	0	—	[44]
Pneumoviridae	*Metapneumovirus*	*Human metapneumovirus*	nonsevere		1	0	0	—	[4]
Pneumoviridae	*Orthopneumovirus*	*Human orthopneumovirus*	nonsevere		1	0	0	—	[2,4]
Rhabdoviridae	*Lyssavirus*	*Australian bat lyssavirus*	severe	Rabies-like disease, CFR 100% (3 cases)	1	1	N/A	0.865	[2]
Rhabdoviridae	*Lyssavirus*	*Duvenhage lyssavirus*	severe	Rabies-like disease, CFR 75% (4 cases)	1	1	N/A	0.862	[1,2]
Rhabdoviridae	*Lyssavirus*	*European bat 1 lyssavirus*	severe	Rabies-like disease, CFR 100% (3 cases)	1	0	N/A	0.880	[2]
Rhabdoviridae	*Lyssavirus*	*European bat 2 lyssavirus*	severe	Rabies-like disease, CFR 20-50%	1	1	N/A	0.877	[1,2]
Rhabdoviridae	*Lyssavirus*	*Irkut lyssavirus*	severe	Rabies-like disease, CFR 100% (1 case)	1	1	N/A	0.849	[45]
Rhabdoviridae	*Lyssavirus*	*Mokola lyssavirus*	severe	Neurologic disease different to classical rabies, CFR 33% (3 cases)	1	0	N/A	0.867	[2,46]
Rhabdoviridae	*Lyssavirus*	*Rabies lyssavirus*	severe	Rabies disease, CFR 60%	1	1	N/A	0.754	[4]
Rhabdoviridae	*Tibrovirus*	*Bas-Congo tibrovirus*	severe	Haemorrhagic fever, CFR 67% (3 cases)	1	0	N/A	—	[47]
Rhabdoviridae	*Tibrovirus*	*Ekpoma 1 tibrovirus*	nonsevere		0	0	0	—	[48]
Rhabdoviridae	*Tibrovirus*	*Ekpoma 2 tibrovirus*	nonsevere		0	0	0	—	[48]
Rhabdoviridae	*Vesiculovirus*	*Alagoas vesiculovirus*	nonsevere		0	0	0	0.050	[1,2,49]
Rhabdoviridae	***Vesiculovirus***	***Chandipura vesiculovirus***	**nonsevere**		1	0	0	**0.879**	[4]

Rhabdoviridae	*Vesiculovirus*	*Cocal vesiculovirus*	nonsevere	0	0	0	0.046	[50]
Rhabdoviridae	*Vesiculovirus*	*Indiana vesiculovirus*	nonsevere	0	0	0	0.048	[1,2,49]
Rhabdoviridae	*Vesiculovirus*	*Isfahan vesiculovirus*	nonsevere	0	0	0	0.046	[1]
Rhabdoviridae	*Vesiculovirus*	*Maraba vesiculovirus*	nonsevere	0	0	0	0.047	[4,51]
Rhabdoviridae	*Vesiculovirus*	*New Jersey vesiculovirus*	nonsevere	0	0	0	0.047	[1,2,49]
Rhabdoviridae	*Vesiculovirus*	*Piry vesiculovirus*	nonsevere	0	0	0	0.051	[1,52]
+ssRNA viruses								
Astroviridae	*Mamastrovirus*	*Mamastrovirus 1*	nonsevere	1	0	0	0.047	[2,4,53]
Astroviridae	*Mamastrovirus*	*Mamastrovirus 6*	nonsevere	0	0	0	0.046	[54–56]
Astroviridae	*Mamastrovirus*	*Mamastrovirus 8*	nonsevere	1	0	1	0.051	[55–58]
Astroviridae	*Mamastrovirus*	*Mamastrovirus 9*	nonsevere	0	0	0	0.043	[55,59]
Caliciviridae	*Norovirus*	*Norwalk virus*	nonsevere	1	0	0	—	[1,4]
Caliciviridae	*Sapovirus*	*Sapporo virus*	nonsevere	0	0	0	—	[4]
Caliciviridae	*Vesivirus*	*Vesicular exanthema of swine virus*	nonsevere	0	0	0	—	[60]
Coronaviridae	*Alphacoronavirus*	*Alphacoronavirus 1*	nonsevere	0	0	0	—	[61]
Coronaviridae	*Alphacoronavirus*	*Human coronavirus 229E*	nonsevere	1	0	0	—	[2,4,62]
Coronaviridae	*Alphacoronavirus*	*Human coronavirus NL63*	nonsevere	1	0	0	—	[1,2,63]
Coronaviridae	*Betacoronavirus*	*Betacoronavirus 1*	nonsevere	1	0	0	—	[2,4,64]
Coronaviridae	*Betacoronavirus*	*Human coronavirus HKU1*	nonsevere	0	0	0	—	[2,4]

Coronaviridae	*Betacoronavirus*	*Middle East respiratory syndrome-related coronavirus*	severe	CFR 27-56%	1	1	N/A	—	[65–67]
Coronaviridae	*Betacoronavirus*	*Severe acute respiratory syndrome-related coronavirus*	severe	CFR 9-12%	1	1	N/A	—	[2,4]
Coronaviridae	*Torovirus*	*Human torovirus*	nonsevere		0	0	0	—	[1,2]
Flaviviridae	*Flavivirus*	*Aroa virus*	nonsevere		0	0	0	0.133	[68]
Flaviviridae	*Flavivirus*	*Bagaza virus*	nonsevere		0	0	0	0.120	[69]
Flaviviridae	*Flavivirus*	*Banzi virus*	nonsevere		0	0	0	0.100	[1,2,4]
Flaviviridae	***Flavivirus***	***Cacipacore virus***	**severe**	Only known case required hospitalisation	0	0	N/A	**0.072**	[70]
Flaviviridae	***Flavivirus***	***Dengue virus***	**nonsevere**		1	1	0	**0.686**	[2,4]
Flaviviridae	*Flavivirus*	*Edge Hill virus*	nonsevere		0	0	0	0.129	[1,71]
Flaviviridae	*Flavivirus*	*Gadgets Gully virus*	nonsevere		0	0	0	0.109	[4]
Flaviviridae	*Flavivirus*	*Ilheus virus*	severe	Described as potentially severe, one subspecies (Rocio virus) has CFR 10%	1	1	N/A	0.777	[1]
Flaviviridae	*Flavivirus*	*Japanese encephalitis virus*	severe	CFR 5-40%	1	1	N/A	0.515	[4]
Flaviviridae	*Flavivirus*	*Kokobera virus*	nonsevere		0	0	0	0.123	[1,4]
Flaviviridae	***Flavivirus***	***Kyasanur forest disease virus***	**severe**	CFR 3-25%	1	1	N/A	**0.197**	[1,2,4]
Flaviviridae	*Flavivirus*	*Langat virus*	nonsevere		0	0	0	0.130	[1,2,4,72]
Flaviviridae	***Flavivirus***	***Louping ill virus***	**nonsevere**		0	0	0	**0.836**	[1,2]
Flaviviridae	*Flavivirus*	*Murray Valley encephalitis virus*	severe	CFR 18-42%, frequent neurologic sequelae	1	1	N/A	0.785	[1,2,4]
Flaviviridae	*Flavivirus*	*Ntaya virus*	nonsevere		0	0	0	0.264	[73]

Flaviviridae	*Flavivirus*	*Omsk hemorrhagic fever virus*	nonsevere		1	1	0	0.455	[74]
Flaviviridae	*Flavivirus*	*Powassan virus*	severe	CFR 10-15%, frequent neurologic sequelae	1	0	N/A	0.780	[1,2,4]
Flaviviridae	***Flavivirus***	***Rio Bravo virus***	**severe**	Two of the five known cases required hospitalisation	0	0	N/A	**0.115**	[4,75]
Flaviviridae	*Flavivirus*	*St. Louis encephalitis virus*	severe	CFR 7-30%	1	1	N/A	0.752	[4]
Flaviviridae	*Flavivirus*	*Tembusu virus*	nonsevere		0	0	0	0.109	[76]
Flaviviridae	*Flavivirus*	*Tick-borne encephalitis virus*	severe	CFR 1-54%	1	1	N/A	0.728	[2,4]
Flaviviridae	*Flavivirus*	*Uganda S virus*	nonsevere		0	0	0	0.128	[2,77]
Flaviviridae	***Flavivirus***	***Usutu virus***	**nonsevere**		0	0	0	**0.811**	[1,4,78,79]
Flaviviridae	*Flavivirus*	*Wesselsbron virus*	nonsevere		0	0	0	0.168	[1,4]
Flaviviridae	***Flavivirus***	***West Nile virus***	**nonsevere**		1	1	0	**0.772**	[4]
Flaviviridae	*Flavivirus*	*Yellow fever virus*	severe	CFR 11-50%, haemorrhagic fever	1	1	N/A	0.506	[1,2,4]
Flaviviridae	*Flavivirus*	*Zika virus*	nonsevere		0	0	0	0.197	[2,4]
Flaviviridae	*Hepacivirus*	*Hepacivirus C*	severe	Chronic disease including cirrhosis	1	1	N/A	—	[4]
Flaviviridae	*Pegivirus*	*Pegivirus A*	nonsevere		0	0	0	—	[80]
Flaviviridae	*Pestivirus*	*Bovine viral diarrhea virus 1*	nonsevere		0	0	0	—	[81]
Hepeviridae	*Orthohepevirus*	*Orthohepevirus A*	nonsevere		1	1	0	—	[1,4]
Picornaviridae	*Aphthovirus*	*Equine rhinitis A virus*	nonsevere		0	0	0	—	[82]
Picornaviridae	*Aphthovirus*	*Foot-and-mouth disease virus*	nonsevere		0	0	0	—	[83]
Picornaviridae	*Cardiovirus*	*Cardiovirus A*	nonsevere		0	0	0	—	[84]

Picornaviridae	*Cardiovirus*	*Cardiovirus B*	nonsevere		1	1	1	—	[4,85–87]
Picornaviridae	*Cosavirus*	*Cosavirus A*	nonsevere		0	0	0	0.046	[88–91]
Picornaviridae	*Cosavirus*	*Cosavirus B*	nonsevere		0	0	0	0.048	[88–90]
Picornaviridae	*Cosavirus*	*Cosavirus D*	nonsevere		0	0	0	0.046	[88–90,92,93]
Picornaviridae	*Cosavirus*	*Cosavirus E*	nonsevere		0	0	0	0.045	[89,94,95]
Picornaviridae	*Cosavirus*	*Cosavirus F*	nonsevere		0	0	0	0.051	[89]
Picornaviridae	*Enterovirus*	*Enterovirus A*	nonsevere		1	1	1	0.048	[1,96]
Picornaviridae	*Enterovirus*	*Enterovirus B*	nonsevere		1	1	1	0.049	[1,4]
Picornaviridae	*Enterovirus*	*Enterovirus C*	nonsevere		1	1	1	0.048	[1,4]
Picornaviridae	*Enterovirus*	*Enterovirus D*	nonsevere		1	0	1	0.046	[4,97]
Picornaviridae	*Enterovirus*	*Enterovirus E*	nonsevere		0	0	0	0.045	[98]
Picornaviridae	*Enterovirus*	*Rhinovirus A*	nonsevere		1	0	0	0.046	[1,4,99]
Picornaviridae	*Enterovirus*	*Rhinovirus B*	nonsevere		0	0	0	0.045	[1,4]
Picornaviridae	*Enterovirus*	*Rhinovirus C*	nonsevere		1	0	0	0.047	[1,4,99]
Picornaviridae	*Erbovirus*	*Erbovirus A*	nonsevere		0	0	0	—	[100]
Picornaviridae	*Hepatovirus*	*Hepatovirus A*	nonsevere		1	1	0	—	[1,4]
Picornaviridae	*Kobuvirus*	*Aichivirus A*	nonsevere		0	0	0	—	[101,102]
Picornaviridae	*Parechovirus*	*Parechovirus A*	nonsevere		1	0	1	—	[2,103–105]
Picornaviridae	*Parechovirus*	*Parechovirus B*	severe	Associated with sudden infant death syndrome and adult myocarditis	0	0	N/A	—	[106–109]

Picornaviridae	*Salivirus*	*Salivirus A*	nonsevere		0	0	0	—	[110–112]
Togaviridae	*Alphavirus*	*Barmah Forest virus*	nonsevere		0	0	0	0.049	[4]
Togaviridae	*Alphavirus*	*Chikungunya virus*	nonsevere		1	1	0	0.044	[2,4]
Togaviridae	*Alphavirus*	*Eastern equine encephalitis virus*	severe	CFR 30-70%	1	1	N/A	—	[2,4]
Togaviridae	***Alphavirus***	***Everglades virus***	**nonsevere**		0	0	0	**0.797**	[1,113]
Togaviridae	*Alphavirus*	*Getah virus*	nonsevere		0	0	0	0.050	[1,4]
Togaviridae	*Alphavirus*	*Highlands J virus*	nonsevere		0	0	0	0.051	[114]
Togaviridae	*Alphavirus*	*Mayaro virus*	nonsevere		0	0	0	0.046	[2]
Togaviridae	*Alphavirus*	*Mosso das Pedras virus*	nonsevere		0	0	0	0.044	[115]
Togaviridae	*Alphavirus*	*Mucambo virus*	nonsevere		0	0	0	0.047	[2,116]
Togaviridae	*Alphavirus*	*Ndumu virus*	nonsevere		0	0	0	0.047	[117]
Togaviridae	*Alphavirus*	*O'nyong-nyong virus*	nonsevere		0	0	0	0.041	[1,4,118]
Togaviridae	*Alphavirus*	*Pixuna virus*	nonsevere		0	0	0	0.043	[116]
Togaviridae	*Alphavirus*	*Rio Negro virus*	nonsevere		0	0	0	0.048	[1,4]
Togaviridae	***Alphavirus***	***Ross River virus***	**nonsevere**		0	0	0	**0.535**	[1,2,4]
Togaviridae	***Alphavirus***	***Semliki Forest virus***	**nonsevere**		0	0	0	**0.695**	[1,4]
Togaviridae	*Alphavirus*	*Sindbis virus*	nonsevere		0	0	0	0.054	[1,2,4]
Togaviridae	*Alphavirus*	*Tonate virus*	nonsevere		1	0	0	0.044	[1,4,119,120]
Togaviridae	*Alphavirus*	*Una virus*	nonsevere		0	0	0	0.048	[2,4]
Togaviridae	***Alphavirus***	***Venezuelan equine encephalitis virus***	**nonsevere**		1	1	1	**0.626**	[1,2,4,113]

Togaviridae	*Alphavirus*	*Western equine encephalitis virus*	severe	CFR 3-10%	1	1	N/A	—	[1,4]
Togaviridae	*Alphavirus*	*Whataroa virus*	nonsevere		0	0	0	0.048	[121]
Togaviridae	*Rubivirus*	*Rubella virus*	nonsevere		1	1	0	—	[1,4]
dsRNA viruses									
Picobirnaviridae	*Picobirnavirus*	*Human picobirnavirus*	nonsevere		0	0	0	—	[2]
Reoviridae	*Coltivirus*	*Colorado tick fever virus*	nonsevere		1	0	0	—	[1]
Reoviridae	*Coltivirus*	*Eyach virus*	nonsevere		0	0	0	—	[122]
Reoviridae	*Orbivirus*	*Corriparta virus*	nonsevere		0	0	0	0.047	[123]
Reoviridae	*Orbivirus*	*Great Island virus*	nonsevere		0	0	0	0.075	[1,124]
Reoviridae	*Orbivirus*	*Lebombo virus*	nonsevere		0	0	0	0.043	[1]
Reoviridae	*Orbivirus*	*Orungo virus*	nonsevere		0	0	0	0.054	[1]
Reoviridae	*Orthoreovirus*	*Mammalian orthoreovirus*	nonsevere		0	0	0	—	[4]
Reoviridae	*Orthoreovirus*	*Nelson Bay orthoreovirus*	nonsevere		0	0	0	—	[125–127]
Reoviridae	*Rotavirus*	*Rotavirus A*	nonsevere		1	0	0	0.047	[1,4]
Reoviridae	*Rotavirus*	*Rotavirus B*	nonsevere		1	0	0	0.045	[4]
Reoviridae	*Rotavirus*	*Rotavirus C*	nonsevere		0	0	0	0.049	[1,4]
Reoviridae	*Rotavirus*	*Rotavirus H*	nonsevere		0	0	0	0.048	[128,129]
Reoviridae	*Seadornavirus*	*Banna virus*	nonsevere		0	0	0	—	[1,130,131]

ssRNA-RT viruses									
Retroviridae	*Deltaretrovirus*	*Primate T-lymphotropic virus 1*	severe	Chronic neoplastic and neurologic disease including lymphoma, leukaemia, and HTLV-associated myelopathy	1	1	N/A	—	[1,4]
Retroviridae	*Deltaretrovirus*	*Primate T-lymphotropic virus 2*	severe	Chronic neurologic disease including HTLV-associated myelopathy	1	1	N/A	—	[1,132]
Retroviridae	*Lentivirus*	*Human immunodeficiency virus 1*	severe	AIDS	1	1	N/A	—	[4]
Retroviridae	*Lentivirus*	*Human immunodeficiency virus 2*	severe	AIDS	1	1	N/A	—	[4]
Retroviridae	*Lentivirus*	*Simian immunodeficiency virus*	nonsevere		0	0	0	—	[133]
Retroviridae	*Spumavirus*	*African green monkey simian foamy virus*	nonsevere		0	0	0	—	[4,134]
Retroviridae	*Spumavirus*	*Macaque simian foamy virus*	nonsevere		0	0	0	—	[4,135,136]
Retroviridae	*Spumavirus*	*Simian foamy virus*	nonsevere		0	0	0	—	[4]

References from Table S1:

1. Richman DD, Whitley RJ, Hayden FG. Clinical virology. John Wiley & Sons; 2009.

2. Zuckerman AJ, Banatvala JE, Griffiths P, Schoub B, Mortimer P. Principles and practice of clinical virology. John Wiley & Sons; 2009.

3. Delgado S, Erickson BR, Agudo R, Blair PJ, Vallejo E, Albariño CG, et al. Chapare virus, a newly discovered arenavirus isolated from a fatal hemorrhagic fever case in Bolivia. PLoS Pathog. 2008;4: e1000047.

4. Knipe DM, Howley PM. Fields virology, 5th Edition. Lippincott Williams & Wilkins; 2007.

5. Briese T, Paweska JT, McMullan LK, Hutchison SK, Street C, Palacios G, et al. Genetic detection and characterization of Lujo virus, a new hemorrhagic fever–associated arenavirus from southern Africa. PLoS Pathog. 2009;5: e1000455. doi:10.1371/journal.ppat.1000455

6. Paweska JT, Sewlall NH, Ksiazek TG, Blumberg LH, Hale MJ, Lipkin WI, et al. Nosocomial outbreak of novel arenavirus infection, Southern Africa. Emerg Infect Dis. 2009;15: 1598–1602. doi:10.3201/eid1510.090211

7. Georges AJ, Gonzalez JP, Abdul-Wahid S, Saluzzo JF, Meunier DMY, McCormick JB. Antibodies to Lassa and lassa-like viruses in man and mammals in the Central African Republic. Trans R Soc Trop Med Hyg. 1985;79: 78–79. doi:10.1016/0035-9203(85)90242-1

8. CDC. Fatal illnesses associated with a new world arenavirus--California, 1999-2000. MMWR Morb Mortal Wkly Rep. 2000;49: 709–711.

9. Hoffmann B, Tappe D, Höper D, Herden C, Boldt A, Mawrin C, et al. A Variegated Squirrel Bornavirus Associated with Fatal Human Encephalitis. N Engl J Med. 2015;373: 154–162. doi:10.1056/NEJMoa1415627

10. Towner JS, Sealy TK, Khristova ML, Albariño CG, Conlan S, Reeder SA, et al. Newly discovered ebola virus associated with hemorrhagic fever outbreak in Uganda. PLoS Pathog. 2008;4: e1000212. doi:10.1371/journal.ppat.1000212

11. MacNeil A, Farnon EC, Wamala J, Okware S, Cannon DL, Reed Z, et al. Proportion of deaths and clinical features in Bundibugyo ebola virus infection, Uganda. Emerg Infect Dis. 2010;16: 1969–1972. doi:10.3201/eid1612.100627

12. Khan AS, Spiropoulou CF, Morzunov S, Zaki SR, Kohn MA, Nawas SR, et al. Fatal illness associated with a new hantavirus in Louisiana. J Med Virol. 1995;46: 281–286.

13. Vincent MJ, Quiroz E, Gracia F, Sanchez AJ, Ksiazek TG, Kitsutani PT, et al. Hantavirus pulmonary syndrome in Panama: identification of novel hantaviruses and their likely reservoirs. Virology. 2000;277: 14–19. doi:10.1006/viro.2000.0563

14. Gracia F, Armien B, Simpson SQ, Munoz C, Broce C, Pascale JM, et al. Convalescent pulmonary dysfunction following hantavirus pulmonary syndrome in Panama and the United States. Lung. 2010;188: 387–391. doi:10.1007/s00408-010-9245-4

15. Nelson R, Cañate R, Pascale JM, Dragoo JW, Armien B, Armien AG, et al. Confirmation of Choclo virus as the cause of hantavirus cardiopulmonary syndrome and high serum antibody prevalence in Panama. J Med Virol. 2010;82: 1586–1593. doi:10.1002/jmv.21864

16. Johnson AM, Bowen MD, Ksiazek TG, Williams RJ, Bryan RT, Mills JN, et al. Laguna Negra virus associated with HPS in western Paraguay and Bolivia. Virology. 1997;238: 115–127.

17. Klempa B, Witkowski PT, Popugaeva E, Auste B, Koivogui L, Fichet-Calvet E, et al. Sangassou Virus, the First Hantavirus Isolate from Africa, Displays Genetic and Functional Properties Distinct from Those of Other Murinae-Associated Hantaviruses. J Virol. 2012;86: 3819–3827. doi:10.1128/JVI.05879-11

18. Pattamadilok S, Lee B-H, Kumperasart S, Yoshimatsu K, Okumura M, Nakamura I, et al. Geographical distribution of hantaviruses in Thailand and potential human health significance of Thailand virus. Am J Trop Med Hyg. 2006;75: 994–1002.

19. Okumura M, Yoshimatsu K, Kumperasart S, Nakamura I, Ogino M, Taruishi M, et al. Development of Serological Assays for Thottapalayam Virus, an Insectivore-Borne Hantavirus. Clin Vaccine Immunol. 2007;14: 173–181. doi:10.1128/CVI.00347-06

20. Klempa B, Meisel H, Räth S, Bartel J, Ulrich R, Krüger DH. Occurrence of renal and pulmonary syndrome in a region of northeast Germany where Tula hantavirus circulates. J Clin Microbiol. 2003;41: 4894–4897. doi:10.1128/JCM.41.10.4894-4897.2003

21. Treib J, Dobler G, Haass A, von Blohn W, Strittmatter M, Pindur G, et al. Thunderclap headache caused by Erve virus? Neurology. 1998;50: 509–511.

22. Woessner R, Grauer MT, Langenbach J, Dobler G, Kroeger J, Mielke HG, et al. The Erve virus: possible mode of transmission and reservoir. Infection. 2000;28: 164–166.

23. Butenko AM, Leshchinskaia EV, Semashko IV, Donets MA, Mart'ianova LI. [Dhori virus--a causative agent of human disease. 5 cases of laboratory infection]. Vopr Virusol. 1987;32: 724–729.

24. Moore DL, Causey OR, Carey DE, Reddy S, Cooke AR, Akinkugbe FM, et al. Arthropod-borne viral infections of man in Nigeria, 1964-1970. Ann Trop Med Parasitol. 1975;69: 49.

25. Goebel SJ, Taylor J, Barr BC, Kiehn TE, Castro-Malaspina HR, Hedvat CV, et al. Isolation of Avian paramyxovirus 1 from a patient with a lethal case of pneumonia. J Virol. 2007;81: 12709–12714. doi:10.1128/JVI.01406-07

26. ProMED-mail | Hendra virus, human, equine - Australia (04): (QL) fatal [Internet]. [cited 3 Jul 2014]. Available: http://www.promedmail.org/direct.php?id=283896

27. Selby PL, Davies M, Mee AP. Canine distemper virus induces human osteoclastogenesis through NF-kappaB and sequestosome 1/P62 activation. J Bone Miner Res Off J Am Soc Bone Miner Res. 2006;21: 1750–1756. doi:10.1359/jbmr.060805

28. Chatziandreou N, Stock N, Young D, Andrejeva J, Hagmaier K, McGeoch DJ, et al. Relationships and host range of human, canine, simian and porcine isolates of simian virus 5 (parainfluenza virus 5). J Gen Virol. 2004;85: 3007–3016. doi:10.1099/vir.0.80200-0

29. Itoh H, Morimoto Y, Doi Y, Sanpe T. Studies on simian viruses. Some properties of SV41 grown in Vero cell cultures and search for serum neutralizing antibodies in humans and various animals. Virus. 1968;18: 495–503.

30. Albarino CG, Foltzer M, Towner JS, Rowe LA, Campbell S, Jaramillo CM, et al. Novel paramyxovirus associated with severe acute febrile disease, South Sudan and Uganda, 2012. Emerg Infect Dis. 2014;20: 211–216. doi:10.3201/eid2002.131620

31. Mackenzie JS, Field HE, Guyatt KJ. Managing emerging diseases borne by fruit bats (flying foxes), with particular reference to henipaviruses and Australian bat lyssavirus. J Appl Microbiol. 2003;94: 59–69. doi:10.1046/j.1365-2672.94.s1.7.x

32. Tauro LB, Almeida FL, Contigiani MS. First detection of human infection by Cache Valley and Kairi viruses (Orthobunyavirus) in Argentina. Trans R Soc Trop Med Hyg. 2009;103: 197–199.

33. Rodaniche E de, Andrade AP, Galindo P. Isolation of two antigenically distinct arthropod-borne viruses of group C in Panama. Am J Trop Med Hyg. 1964;13: 839–843.

34. Causey OR, Causey CE, Maroja OM, Macedo DG. The isolation of arthropod-borne viruses, including members of two hitherto undescribed serological groups, in the Amazon region of Brazil. Am J Trop Med Hyg. 1961;10: 227–249.

35. Digoutte JP, Gagnard VJM, Brès P, Pajot F-X. Infection à virus Nyando chez l'homme. Bull Société Pathol Exot. 1972;65: 751–758.

36. Scherer WF, Anderson K, Dickerman RW, Ordonez JV. Studies of Patois Group Arboviruses in Mexico, Guatemala, Honduras, and British Honduras. Am J Trop Med Hyg. 1972;21: 194–200.

37. Vasconcelos PFC, Rosa A, Rosa J, Dégallier N. Concomitant infections by malaria and arboviruses in the Brazilian Amazon region. Rev Latinoam Microbiol. 1990;32: 291–294.

38. Srihongse S, Johnson CM. Wyeomyia subgroup of arbovirus: isolation from man. Science. 1965;149: 863–864.

39. Palacios G, Tesh R, Travassos da Rosa A, Savji N, Sze W, Jain K, et al. Characterization of the Candiru antigenic complex (Bunyaviridae: Phlebovirus), a highly diverse and reassorting group of viruses affecting humans in tropical America. J Virol. 2011;85: 3811–3820. doi:10.1128/JVI.02275-10

40. Yu X-J, Liang M-F, Zhang S-Y, Liu Y, Li J-D, Sun Y-L, et al. Fever with thrombocytopenia associated with a novel bunyavirus in China. N Engl J Med. 2011;364: 1523–1532. doi:10.1056/NEJMoa1010095

41. Xie Q, Li X, Cheng J, Shao Y. Multiple organ damage caused by a novel tick-borne bunyavirus: A case report. J Vector Borne Dis. 2013;50: 314–317.

42. Zhang Y-Z, Zhou D-J, Xiong Y, Chen X-P, He Y-W, Sun Q, et al. Hemorrhagic fever caused by a novel tick-borne Bunyavirus in Huaiyangshan, China. Zhonghua Liu Xing Bing Xue Za Zhi Zhonghua Liuxingbingxue Zazhi. 2011;32: 209–220.

43. Zhao L, Zhai S, Wen H, Cui F, Chi Y, Wang L, et al. Severe fever with thrombocytopenia syndrome virus, Shandong Province, China. Emerg Infect Dis. 2012;18: 963–965. doi:10.3201/eid1806.111345

44. Kayali G, Ortiz EJ, Chorazy ML, Nagaraja KV, DeBeauchamp J, Webby RJ, et al. Serologic evidence of avian metapneumovirus infection among adults occupationally exposed to Turkeys. Vector Borne Zoonotic Dis Larchmt N. 2011;11: 1453–1458. doi:10.1089/vbz.2011.0637

45. Leonova GN, Belikov SI, Kondratov IG, Krylova NV, Pavlenko EV, Romanova EV, et al. A fatal case of bat lyssavirus infection in Primorye Territory of the Russian Far East. Rabies Bull Eur. 2009;33: 5–8.

46. Familusi JB, Osunkoya BO, Moore DL, Kemp GE, Fabiyi A. A fatal human infection with Mokola virus. Am J Trop Med Hyg. 1972;21: 959–963.

47. Grard G, Fair JN, Lee D, Slikas E, Steffen I, Muyembe J-J, et al. A novel rhabdovirus associated with acute hemorrhagic fever in Central Africa. PLoS Pathog. 2012;8: e1002924. doi:10.1371/journal.ppat.1002924

48. Stremlau MH, Andersen KG, Folarin OA, Grove JN, Odia I, Ehiane PE, et al. Discovery of novel rhabdoviruses in the blood of healthy individuals from West Africa. PLoS Negl Trop Dis. 2015;9: e0003631. doi:10.1371/journal.pntd.0003631

49. Letchworth GJ, Rodriguez LL, Del Cbarrera J. Vesicular stomatitis. Vet J. 1999;157: 239–260.

50. Jonkers AH, Shope RE, Aitken TH, Spence L. COCAL VIRUS, A NEW AGENT IN TRINIDAD RELATED TO VESICULAR STOMATITIS VIRUS, TYPE INDIANA. Am J Vet Res. 1964;25: 236–242.

51. Travassos da Rosa APA, Tesh RB, Travassos da Rosa JF, Herve JP, Main AJJ. Carajas and Maraba viruses, two new vesiculoviruses isolated from phlebotomine sand flies in Brazil. Am J Trop Med Hyg. 1984;33: 999–1006.

52. Pedrosa PBS, Cardoso TAO. Viral infections in workers in hospital and research laboratory settings: a comparative review of infection modes and respective biosafety aspects. Int J Infect Dis. 2011;15: e366–e376. doi:10.1016/j.ijid.2011.03.005

53. Singh PB, Sreenivasan MA, Pavri KM. Viruses in acute gastroenteritis in children in Pune, India. Epidemiol Infect. 1989;102: 345–353.

54. Wylie KM, Mihindukulasuriya KA, Sodergren E, Weinstock GM, Storch GA. Sequence analysis of the human virome in febrile and afebrile children. PLoS ONE. 2012;7: e27735. doi:10.1371/journal.pone.0027735

55. Guix S, Bosch A, Pintó RM. Astrovirus Taxonomy. In: Schultz-Cherry S, editor. Astrovirus Research. Springer New York; 2013. pp. 97–118.

56. Finkbeiner SR, Holtz LR, Jiang Y, Rajendran P, Franz CJ, Zhao G, et al. Human stool contains a previously unrecognized diversity of novel astroviruses. Virol J. 2009;6: 161. doi:10.1186/1743-422X-6-161

57. Ahmed SF, Sebeny PJ, Klena JD, Pimentel G, Mansour A, Naguib AM, et al. Novel astroviruses in children, Egypt. Emerg Infect Dis. 2011;17: 2391–2393. doi:10.3201/eid1712.110909

58. Phan TG, Vo NP, Bonkoungou IJO, Kapoor A, Barro N, O'Ryan M, et al. Acute diarrhea in West African children: diverse enteric viruses and a novel parvovirus genus. J Virol. 2012;86: 11024–11030. doi:10.1128/JVI.01427-12

59. Finkbeiner SR, Li Y, Ruone S, Conrardy C, Gregoricus N, Toney D, et al. Identification of a novel astrovirus (Astrovirus VA1) associated with an outbreak of acute gastroenteritis. J Virol. 2009;83: 10836–10839. doi:10.1128/JVI.00998-09

60. Smith AW, Berry ES, Skilling DE, Barlough JE, Poet SE, Berke T, et al. In vitro isolation and characterization of a calicivirus causing a vesicular disease of the hands and feet. Clin Infect Dis Off Publ Infect Dis Soc Am. 1998;26: 434–439.

61. Terao Y, Takagi H, Phan TG, Okitsu S, Ushijima H. Identification of antibody against porcine coronavirus in human milk. Clin Lab. 2007;53: 129–130.

62. Pene F, Merlat A, Vabret A, Rozenberg F, Buzyn A, Dreyfus F, et al. Coronavirus 229E-Related Pneumonia in Immunocompromised Patients. Clin Infect Dis. 2003;37: 929–932. doi:10.1086/377612

63. Bastien N, Anderson K, Hart L, Caeseele PV, Brandt K, Milley D, et al. Human coronavirus NL63 infection in Canada. J Infect Dis. 2005;191: 503–506. doi:10.1086/426869

64. Patrick DM, Petric M, Skowronski DM, Guasparini R, Booth TF, Krajden M, et al. An outbreak of Human coronavirus OC43 infection and serological cross-reactivity with SARS coronavirus. Can J Infect Dis Med Microbiol. 2006;17: 330–336.

65. Zaki AM, Van Boheemen S, Bestebroer TM, Osterhaus AD, Fouchier RA. Isolation of a novel coronavirus from a man with pneumonia in Saudi Arabia. N Engl J Med. 2012;367: 1814–1820.

66. WHO. Frequently asked questions on Middle East respiratory syndrome coronavirus (MERS-CoV) [Internet]. [cited 10 Sep 2014]. Available: http://www.who.int/csr/disease/coronavirus_infections/faq/en/

67. Chan JF-W, Lau SK-P, Woo PC-Y. The emerging novel Middle East respiratory syndrome coronavirus: the "knowns" and "unknowns." J Formos Med Assoc Taiwan Yi Zhi. 2013;112: 372–381. doi:10.1016/j.jfma.2013.05.010

68. Srihongse S, Johnson CM. The first isolation of Bussuquara virus from man. Trans R Soc Trop Med Hyg. 1971;65: 541–542.

69. Calisher CH, Gould EA. Taxonomy of the virus family Flaviviridae. Advances in Virus Research. Academic Press; 2003. pp. 1–19.

70. Batista WC, Tavares G da SB, Vieira DS, Honda ER, Pereira SS, Tada MS. Notification of the first isolation of Cacipacore virus in a human in the State of Rondônia, Brazil. Rev Soc Bras Med Trop. 2011;44: 528–530.

71. Aaskov JG, Phillips DA, Wiemers MA. Possible clinical infection with Edge Hill virus. Trans R Soc Trop Med Hyg. 1993;87: 452–453.

72. Maffioli C, Grandgirard D, Leib SL, Engler O. SiRNA inhibits replication of Langat virus, a member of the Tick-borne encephalitis virus complex in organotypic rat brain slices. PLoS ONE. 2012;7: e44703. doi:10.1371/journal.pone.0044703

73. Woodruff AW, Bowen ET, Platt GS. Viral infections in travellers from tropical Africa. Br Med J. 1978;1: 956–958.

74. Růžek D, Yakimenko VV, Karan LS, Tkachev SE. Omsk haemorrhagic fever. The Lancet. 2010;376: 2104–2113. doi:10.1016/S0140-6736(10)61120-8

75. Sulkin SE, Burns KF, Shelton DF, Wallis C. Bat salivary gland virus: infections of man and monkey. Tex Rep Biol Med. 1962;20: 113.

76. Wolfe ND, Kilbourn AM, Karesh WB, Rahman HA, Bosi EJ, Cropp BC, et al. Sylvatic transmission of arboviruses among Bornean orangutans. Am J Trop Med Hyg. 2001;64: 310–316.

77. Weissenböck H, Hubálek Z, Bakonyi T, Nowotny N. Zoonotic mosquito-borne flaviviruses: worldwide presence of agents with proven pathogenicity and potential candidates of future emerging diseases. Vet Microbiol. 2010;140: 271–280.

78. Cavrini F, Gaibani P, Longo G, Pierro AM, Rossini G, Bonilauri P, et al. Usutu virus infection in a patient who underwent orthotropic liver transplantation, Italy, August-September 2009. Euro Surveill Bull Eur Sur Mal Transm Eur Commun Dis Bull. 2009;14: 19448.

79. Pecorari M, Longo G, Gennari W, Grottola A, Sabbatini AM, Tagliazucchi S, et al. First human case of Usutu virus neuroinvasive infection, Italy, August-September 2009. Eurosurveillance. 2009;14: 19446.

80. Bhattarai N, Stapleton JT. GB virus C: the good boy virus? Trends Microbiol. 2012;20: 124–130. doi:10.1016/j.tim.2012.01.004

81. Giangaspero M, Wellemans G, Vanopdenbosch E, Belloli A, Verhulst A. Bovine viral diarrhoea. The Lancet. 1988;332: 110. doi:10.1016/S0140-6736(88)90047-5

82. Plummer G. An equine respiratory virus with enterovirus properties. Nature. 1962;195: 519–520. doi:10.1038/195519a0

83. Bauer K. Foot- and-mouth disease as zoonosis. Arch Virol Suppl. 1997;13: 95–97.

84. Oberste MS, Gotuzzo E, Blair P, Nix WA, Ksiazek TG, Comer JA, et al. Human febrile illness caused by Encephalomyocarditis virus infection, Peru. Emerg Infect Dis. 2009;15: 640–646. doi:10.3201/eid1504.081428

85. Lipton HL. Human Vilyuisk encephalitis. Rev Med Virol. 2008;18: 347–352. doi:10.1002/rmv.585

86. Goldfarb LG, Vladimirtsev VA, Platonov FA, Lee H-S, McLean CA, Masters CL. Viliuisk encephalomyelitis in Eastern Siberia - analysis of 390 cases: In memory of D Carleton Gajdusek. Folia Neuropathol Assoc Pol Neuropathol Med Res Cent Pol Acad Sci. 2009;47: 171–181.

87. Zoll J, Erkens Hulshof S, Lanke K, Verduyn Lunel F, Melchers WJG, Schoondermark-van de Ven E, et al. Saffold virus, a human Theiler's-like cardiovirus, is ubiquitous and causes infection early in life. PLoS Pathog. 2009;5: e1000416. doi:10.1371/journal.ppat.1000416

88. Kapoor A, Li L, Victoria J, Oderinde B, Mason C, Pandey P, et al. Multiple novel astrovirus species in human stool. J Gen Virol. 2009;90: 2965–2972. doi:10.1099/vir.0.014449-0

89. Kapusinszky B, Phan TG, Kapoor A, Delwart E. Genetic diversity of the genus Cosavirus in the family Picornaviridae: a new species, recombination, and 26 new genotypes. PloS One. 2012;7: e36685. doi:10.1371/journal.pone.0036685

90. Stocker A, Souza BF de CD, Ribeiro TCM, Netto EM, Araujo LO, Correa JI, et al. Cosavirus infection in persons with and without gastroenteritis, Brazil. Emerg Infect Dis. 2012;18: 656–659. doi:10.3201/eid1804.111415

91. Dai XQ, Hua XG, Shan TL, Delwart E, Zhao W. Human cosavirus infections in children in China. J Clin Virol. 2010;48: 228–229. doi:10.1016/j.jcv.2010.03.024

92. Khamrin P, Chaimongkol N, Malasao R, Suantai B, Saikhruang W, Kongsricharoern T, et al. Detection and molecular characterization of cosavirus in adults with diarrhea, Thailand. Virus Genes. 2012;44: 244–246. doi:10.1007/s11262-011-0700-y

93. Cotten M, Oude Munnink B, Canuti M, Deijs M, Watson SJ, Kellam P, et al. Full genome virus detection in fecal samples using sensitive nucleic acid preparation, deep sequencing, and a novel iterative sequence classification algorithm. PLoS ONE. 2014;9: e93269. doi:10.1371/journal.pone.0093269

94. Campanini G, Rovida F, Meloni F, Cascina A, Ciccocioppo R, Piralla A, et al. Persistent human cosavirus infection in lung transplant recipient, Italy. Emerg Infect Dis. 2013;19: 1667–1669. doi:10.3201/eid1910.130352

95. Holtz LR, Finkbeiner SR, Kirkwood CD, Wang D. Identification of a novel picornavirus related to cosaviruses in a child with acute diarrhea. Virol J. 2008;5: 159. doi:10.1186/1743-422X-5-159

96. Yi L, Lu J, Kung H, He M-L. The virology and developments toward control of human enterovirus 71. Crit Rev Microbiol. 2011;37: 313–327. doi:10.3109/1040841X.2011.580723

97. Imamura T. Clusters of acute respiratory illness associated with human enterovirus 68--Asia, Europe, and United States, 2008-2010. MMWR Morb Mortal Wkly Rep. 2011;60: 1301–1304.

98. Gür S, Yapkiç O, Yilmaz A. Serological survey of bovine enterovirus type 1 in different mammalian species in Turkey. Zoonoses Public Health. 2008;55: 106–111. doi:10.1111/j.1863-2378.2007.01095.x

99. Louie JK, Yagi S, Nelson FA, Kiang D, Glaser CA, Rosenberg J, et al. Rhinovirus outbreak in a long term care facility for elderly persons associated with unusually high mortality. Clin Infect Dis Off Publ Infect Dis Soc Am. 2005;41: 262–265. doi:10.1086/430915

100. Kriegshäuser G, Deutz A, Kuechler E, Skern T, Lussy H, Nowotny N. Prevalence of neutralizing antibodies to Equine rhinitis A and B virus in horses and man. Vet Microbiol. 2005;106: 293–296.

101. Ambert-Balay K, Lorrot M, Bon F, Giraudon H, Kaplon J, Wolfer M, et al. Prevalence and genetic diversity of Aichi virus strains in stool samples from community and hospitalized patients. J Clin Microbiol. 2008;46: 1252–1258. doi:10.1128/JCM.02140-07

102. Kaikkonen S, Räsänen S, Rämet M, Vesikari T. Aichi virus infection in children with acute gastroenteritis in Finland. Epidemiol Infect. 2010;138: 1166–1171. doi:10.1017/S0950268809991300

103. Benschop KSM, Schinkel J, Minnaar RP, Pajkrt D, Spanjerberg L, Kraakman HC, et al. Human parechovirus infections in Dutch children and the association between serotype and disease severity. Clin Infect Dis. 2006;42: 204–210. doi:10.1086/498905

104. Ito M, Yamashita T, Tsuzuki H, Takeda N, Sakae K. Isolation and identification of a novel human parechovirus. J Gen Virol. 2004;85: 391–398.

105. Abed Y, Boivin G. Human Parechovirus Infections in Canada. Emerg Infect Dis. 2006;12: 969–975. doi:10.3201/eid1206.051675

106. Blixt M, Sandler S, Bo Niklasson M. D. PD. Ljungan Virus and Diabetes. In: Taylor K, Hyöty H, Toniolo A, Zuckerman AJ, editors. Diabetes and Viruses. Springer New York; 2013. pp. 81–86.

107. Krous HF, Langlois NE. Ljungan virus: a commentary on its association with fetal and infant morbidity and mortality in animals and humans. Birt Defects Res A Clin Mol Teratol. 2010;88: 947–952. doi:10.1002/bdra.20728

108. Samsioe A, Papadogiannakis N, Hultman T, Sjöholm Å, Klitz W, Niklasson B. Ljungan virus present in intrauterine fetal death diagnosed by both immunohistochemistry and PCR. Birt Defects Res A Clin Mol Teratol. 2009;85: 227–229. doi:10.1002/bdra.20554

109. Niklasson B, Samsioe A, Papadogiannakis N, Gustafsson S, Klitz W. Zoonotic Ljungan virus associated with central nervous system malformations in terminated pregnancy. Birt Defects Res A Clin Mol Teratol. 2009;85: 542–545. doi:10.1002/bdra.20568

110. Greninger AL, Runckel C, Chiu CY, Haggerty T, Parsonnet J, Ganem D, et al. The complete genome of klassevirus—a novel picornavirus in pediatric stool. Virol J. 2009;6: 82.

111. Li L, Victoria J, Kapoor A, Blinkova O, Wang C, Babrzadeh F, et al. A novel picornavirus associated with gastroenteritis. J Virol. 2009;83: 12002–12006. doi:10.1128/JVI.01241-09

112. Shan T, Wang C, Cui L, Yu Y, Delwart E, Zhao W, et al. Picornavirus salivirus/klassevirus in children with diarrhea, China. Emerg Infect Dis. 2010;16: 1303–1305. doi:10.3201/eid1608.100087

113. Ehrenkranz NJ, Ventura AK. Venezuelan equine encephalitis virus infection in man. Annu Rev Med. 1974;25: 9–14. doi:10.1146/annurev.me.25.020174.000301

114. Meehan PJ, Wells DL, Paul W, Buff E, Lewis A, Muth D, et al. Epidemiological features of and public health response to a St. Louis encephalitis epidemic in Florida, 1990-1. Epidemiol Infect. 2000;125: 181–188. doi:10.2307/3864999

115. Pisano MB, Oria G, Beskow G, Aguilar J, Konigheim B, Cacace ML, et al. Venezuelan Equine Encephalitis Viruses (VEEV) in Argentina: Serological Evidence of Human Infection. PLoS Negl Trop Dis. 2013;7: e2551. doi:10.1371/journal.pntd.0002551

116. Vasconcelos PF da C, Travassos da Rosa JFS, Travassos da Rosa AP de A, Dégallier N, Pinheiro F de P, Sá Filho GC. Epidemiology of encephalitis by arboviruses in the Amazon region of Brazil. Rev Inst Med Trop São Paulo. 1991;33: 465–476. doi:10.1590/S0036-46651991000600007

117. Kokernot RH, McIntosh BM, Worth CB. Ndumu virus, a hitherto unknown agent, isolated from culicine mosouitoes collected in northern Natal. Union of South Africa. Am J Trop Med Hyg. 1961;10: 383–386.

118. Kiwanuka N, Sanders EJ, Rwaguma EB, Kawamata J, Ssengooba FP, Najjemba R, et al. O'nyong-nyong fever in south-central Uganda, 1996—1997: clinical features and validation of a clinical case definition for surveillance purposes. Clin Infect Dis. 1999;29: 1243–1250. doi:10.1086/313462

119. Hommel D, Heraud JM, Hulin A, Talarmin A. Association of Tonate virus (subtype IIIB of the Venezuelan equine encephalitis complex) with encephalitis in a human. Clin Infect Dis. 2000;30: 188–190. doi:10.1086/313611

120. Talarmin A, Trochu J, Gardon J, Laventure S, Hommel D, Lelarge J, et al. Tonate virus infection in French Guiana: clinical aspects and seroepidemiologic study. Am J Trop Med Hyg. 2001;64: 274–279.

121. Maguire T, Miles JAR, Casals J. Whataroa virus, a group A arbovirus isolated in South Westland, New Zealand. Am J Trop Med Hyg. 1967;16: 371–373.

122. Dobler G. Arboviruses causing neurological disorders in the central nervous system. Arch Virol Suppl. 1996;11: 33–40.

123. Boughton CR, Hawkes RA, Naim HM. Arbovirus infection in humans in NSW: seroprevalence and pathogenicity of certain Australian bunyaviruses. Aust N Z J Med. 1990;20: 51–55.

124. Libíková H, Heinz F, Ujházyová D, Stünzner D. Orbiviruses of the Kemerovo complex and neurological diseases. Med Microbiol Immunol (Berl). 1978;166: 255–263. doi:10.1007/BF02121159

125. Chua KB, Crameri G, Hyatt A, Yu M, Tompang MR, Rosli J, et al. A previously unknown reovirus of bat origin is associated with an acute respiratory disease in humans. Proc Natl Acad Sci. 2007;104: 11424–11429. doi:10.1073/pnas.0701372104

126. Chua KB, Voon K, Crameri G, Tan HS, Rosli J, McEachern JA, et al. Identification and characterization of a new orthoreovirus from patients with acute respiratory infections. PLoS ONE. 2008;3: e3803. doi:10.1371/journal.pone.0003803

127. Chua KB, Voon K, Yu M, Keniscope C, Abdul Rasid K, Wang L-F. Investigation of a potential zoonotic transmission of orthoreovirus associated with acute influenza-like illness in an adult patient. PLoS ONE. 2011;6: e25434. doi:10.1371/journal.pone.0025434

128. Yang H, Chen S, Ji S. [A novel rotavirus causing large scale of adult diarrhea in Shi Jiazhuang]. Zhonghua Liu Xing Bing Xue Za Zhi Zhonghua Liuxingbingxue Zazhi. 1998;19: 336–338.

129. Ji S, Bi Y, Yang H, Yang F, Song J, Tao X, et al. [Cultivation and serial propagation of a new rotavirus causing adult diarrhea in primary human embryo kidney cells]. Zhonghua Yi Xue Za Zhi. 2002;82: 14–18.

130. Liu H, Li MH, Zhai YG, Meng WS, Sun XH, Cao YX, et al. Banna Virus, China, 1987–2007. Emerg Infect Dis. 2010;16: 514.

131. Liu H, Gao X, Liang G. Newly recognized mosquito-associated viruses in mainland China, in the last two decades. Virol J. 2011;8: 68.

132. Araujo A, Hall WW. Human T-lymphotropic virus type II and neurological disease. Ann Neurol. 2004;56: 10–19. doi:10.1002/ana.20126

133. Khabbaz RF, Heneine W, George JR, Parekh B, Rowe T, Woods T, et al. Infection of a Laboratory Worker with Simian Immunodeficiency Virus. N Engl J Med. 1994;330: 172–177. doi:10.1056/NEJM199401203300304

134. Heneine W, Switzer WM, Sandstrom P, Brown J, Vedapuri S, Schable CA, et al. Identification of a human population infected with simian foamy viruses. Nat Med. 1998;4: 403–407. doi:10.1038/nm0498-403

135. Brooks JI, Rud EW, Pilon RG, Smith JM, Switzer WM, Sandstrom PA. Cross-species retroviral transmission from macaques to human beings. The Lancet. 2002;360: 387–388. doi:10.1016/S0140-6736(02)09597-1

136. Jones-Engel L, Engel GA, Schillaci MA, Rompis A, Putra A, Suaryana KG, et al. Primate-to-human retroviral transmission in Asia. Emerg Infect Dis. 2005;11: 1028–1035. doi:10.3201/eid1107.040957
