# Supplementary material for: Tissue tropism and transmission ecology predict virulence of human RNA viruses
Source: PLoS Biol. 2019 Nov 26;17(11):e3000206. doi: 10.1371/journal.pbio.3000206 (PMC6879112; doi:10.1371/journal.pbio.3000206)
Supplement: S2 Table — Partial dependence given as mean marginal relative change in log-odds and mean predicted probability of classifying virulence as ‘severe’ for all predictor variables from random forest models featuring all viruses and models featuring zoonotic viruses only. (PDF) [file pbio.3000206.s002.pdf]

Predictor	Trait	Mean PD (all viruses)	Mean PD (zoonotic)	Mean p(severe) (all viruses)	Mean p(severe) (zoonotic)
**Family**	*Arenaviridae*	-0.243	-0.350	0.391	0.357
	Bornaviridae	-0.188	-0.251	0.409	0.389
	Filoviridae	-0.145	-0.253	0.419	0.385
	Hantaviridae	-0.035	-0.106	0.454	0.432
	Nairoviridae	-0.453	-0.511	0.348	0.322
	Orthomyxoviridae	-0.627	-0.797	0.269	0.233
	Paramyxoviridae	-0.356	-0.664	0.363	0.282
	Peribunyaviridae	-1.157	-1.183	0.305	0.275
	Phenuiviridae	-0.500	-0.551	0.329	0.302
	Pneumoviridae	-0.311	-0.603	0.378	0.295
	Rhabdoviridae	-0.529	-0.494	0.361	0.344
	Astroviridae	0.030	-0.535	0.471	0.320
	Caliciviridae	-0.278	-0.620	0.384	0.295
	Coronaviridae	-0.171	-0.587	0.414	0.304
	Flaviviridae	-0.583	-0.572	0.341	0.320
	Hepeviridae	-0.237	-0.317	0.397	0.368
	Picornaviridae	-0.585	-1.265	0.304	0.244
	Togaviridae	-1.179	-1.154	0.281	0.245
	Picobirnaviridae	0.030	-0.340	0.471	0.363
	Reoviridae	-0.844	-0.996	0.254	0.219
	Retroviridae	-0.295	-0.228	0.381	0.396
**Genome type**	(-)ssRNA	-0.802	-1.015	0.322	0.285
	(+)ssRNA	-0.834	-1.237	0.311	0.262
	dsRNA	-0.868	-1.014	0.282	0.248
	ssRNA-RT	-0.749	-0.672	0.296	0.283
**Transmissibility level**	2	-1.101	-1.311	0.310	0.271
	3	-0.351	-0.418	0.358	0.337
	4	-0.520	-1.033	0.327	0.269
**Transmission: primary route**	direct contact	-0.250	-0.546	0.381	0.312
	faecal-oral	-1.220	-1.442	0.265	0.233
	respiratory	-0.122	-0.377	0.421	0.345
	vector	-1.197	-1.380	0.276	0.245
**Transmission: direct contact**	0	-1.092	-1.517	0.307	0.266
	1	-0.544	-0.723	0.328	0.287
**Transmission: faecal-oral**	0	-1.039	-1.255	0.315	0.276
	1	-0.716	-1.073	0.310	0.265
**Transmission: respiratory**	0	-1.071	-1.506	0.310	0.271
	1	-0.355	-0.631	0.352	0.293
**Transmission: vector**	0	-0.265	-0.827	0.378	0.302
	1	-1.187	-1.373	0.281	0.250
**Transmission: multiple routes**	0	-1.025	-1.451	0.318	0.279
	1	-0.809	-1.031	0.300	0.256
**Transmission: food-borne**	0	-1.050	-1.545	0.313	0.271
	1	-0.548	-0.824	0.329	0.280
**Transmission: vertical**	0	-1.047	-1.504	0.313	0.269
	1	-0.528	-0.601	0.327	0.300
**Tropism: primary**	circulatory	-0.755	-0.613	0.269	0.279
	gastrointestinal	-1.335	-1.654	0.237	0.202
	hepatic	-1.312	-0.665	0.241	0.268
	neural	-0.069	-0.158	0.450	0.417
	respiratory	-0.729	-1.098	0.269	0.208
	systemic	-0.103	-0.186	0.429	0.402
	vascular	-0.701	-0.783	0.270	0.240
	viraemic	-1.291	-1.439	0.246	0.215

**Tropism: vascular**	0	-1.045	-1.501	0.316	0.273
	1	-0.584	-0.695	0.318	0.283
**Tropism: circulatory**	0	-1.045	-1.502	0.314	0.271
	1	-0.803	-0.738	0.310	0.281
**Tropism: gastrointestinal**	0	-1.080	-1.312	0.310	0.270
	1	-0.470	-0.939	0.339	0.286
**Tropism: hepatic**	0	-1.086	-1.532	0.307	0.266
	1	-0.416	-0.520	0.347	0.314
**Tropism: neural**	0	-1.217	-1.624	0.272	0.239
	1	-0.321	-0.508	0.370	0.323
**Tropism: respiratory**	0	-1.110	-1.496	0.305	0.270
	1	-0.498	-0.815	0.330	0.276
**Tropism: cardiac**	0	-1.052	-1.502	0.312	0.271
	1	-0.427	-0.669	0.344	0.289
**Tropism: joints**	0	-1.048	-1.499	0.313	0.272
	1	-0.760	-0.974	0.311	0.267
**Tropism: renal**	0	-1.146	-1.576	0.294	0.254
	1	-0.163	-0.193	0.407	0.396
**Tropism: reproductive**	0	-1.049	-1.498	0.313	0.272
	1	-0.668	-0.927	0.315	0.268
**Tropism: sensory**	0	-1.054	-1.501	0.312	0.272
	1	-0.537	-0.830	0.327	0.275
**Tropism: skin**	0	-1.052	-1.503	0.312	0.271
	1	-0.538	-0.687	0.328	0.287
**Tropism: muscular**	0	-1.072	-1.521	0.311	0.270
	1	-0.467	-0.611	0.339	0.299
**Tropism: endocrine**	0	-1.069	-1.521	0.310	0.267
	1	-0.366	-0.428	0.356	0.332
**Tropism: multiple tropisms**	0	-1.073	-1.513	0.311	0.270
	1	-0.549	-0.720	0.324	0.285
**Host range**	broad	-1.045	-1.288	0.314	0.276
	narrow	-0.785	-1.235	0.307	0.252
**Host: human only**	0	-1.049	-1.337	0.313	0.271
	1	-1.049	-1.075	0.313	0.272
**Host: non-human primates**	0	-0.933	-1.243	0.319	0.284
	1	-1.003	-1.401	0.297	0.249
**Host: other mammal**	0	-1.011	-1.284	0.297	0.244
	1	-1.034	-1.259	0.316	0.279
**Host: bird**	0	-1.203	-1.643	0.298	0.262
	1	-0.502	-0.676	0.336	0.291
