# Supplementary material for: Tissue tropism and transmission ecology predict virulence of human RNA viruses
Source: PLoS Biol. 2019 Nov 26;17(11):e3000206. doi: 10.1371/journal.pbio.3000206 (PMC6879112; doi:10.1371/journal.pbio.3000206)
Supplement: S4 Table — Six-rank system of classifying human RNA virus virulence with available data (specifically, severity rating from main text, fatalities in vulnerable individuals and healthy adults, and severe strains), along with example viruses and number of viruses fitting each exclusive rank’s criteria. (PDF) [file pbio.3000206.s004.pdf]

Rank	Definition	Example virus species	No. virus species
1	Fits any of ‘severe’ criteria outlined in main text (≥5% case fatality ratio, frequent reports of hospitalisation, significant morbidity from certain symptoms, otherwise explicitly described as “severe” or causing “severe disease”)	*Rabies virus*	58
2	Those not fitting ‘severe’ criteria, but are reported to have caused fatalities in healthy adults	*Dengue virus*	14
3	Those not fitting ‘severe’ criteria, but have severe strains or subspecies reported to cause fatalities in healthy adults	*Influenza A virus*	6
4	Those not fitting ‘severe’ criteria, but are reported to have caused fatalities in vulnerable individuals (age 16 and below or 60 and above, immunosuppressed, having co-morbidities, or otherwise ‘at-risk’)	*Rotavirus A*	17
5	Those not fitting ‘severe’ criteria, but have severe strains or subspecies reported to cause fatalities in vulnerable individuals	*Parechovirus A*	3
6	Those not fitting ‘severe’ criteria that have never been reported to cause fatalities	*Human respirovirus 1*	114
