# Supplementary figures and images for: Tissue tropism and transmission ecology predict virulence of human RNA viruses
Source: PLoS Biol. 2019 Nov 26;17(11):e3000206. doi: 10.1371/journal.pbio.3000206 (PMC6879112; doi:10.1371/journal.pbio.3000206)

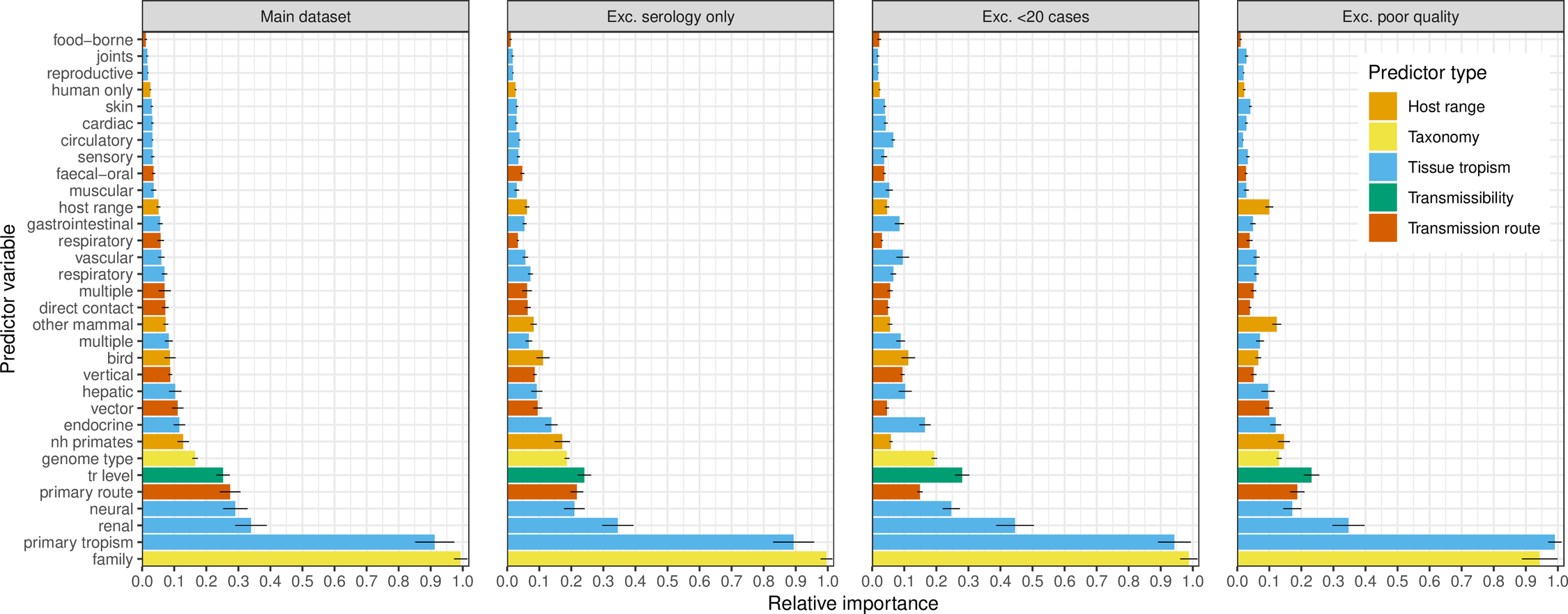

Supplement: S1 Fig — Variable importance for virulence risk factors from random forest models applied to data sets, excluding a) viruses only known to infect humans from serological evidence (n = 36), b) viruses with <20 recognised human infections (n = 55), and c) viruses with poor data quality in at least one predictor (n = 71). Variable importance is calculated as the relative mean decrease in Gini impurity scaled against the most informative predictor within each model alongside importance from the main analysis for comparison. Points denote mean values across 200 training/test partitions. Error bars denote ± 1 standard deviation. Colour key denotes type of predictor variable. Supporting data are available via figshare: 10.6084/m9.figshare.7406441.v3 (https://figshare.com/articles/Data_and_supporting_R_script_for_Tissue_Tropism_and_Transmission_Ecology_Predict_Virulence_of_Human_RNA_Viruses/7406441/3). (TIF) [file pbio.3000206.s006.tif]

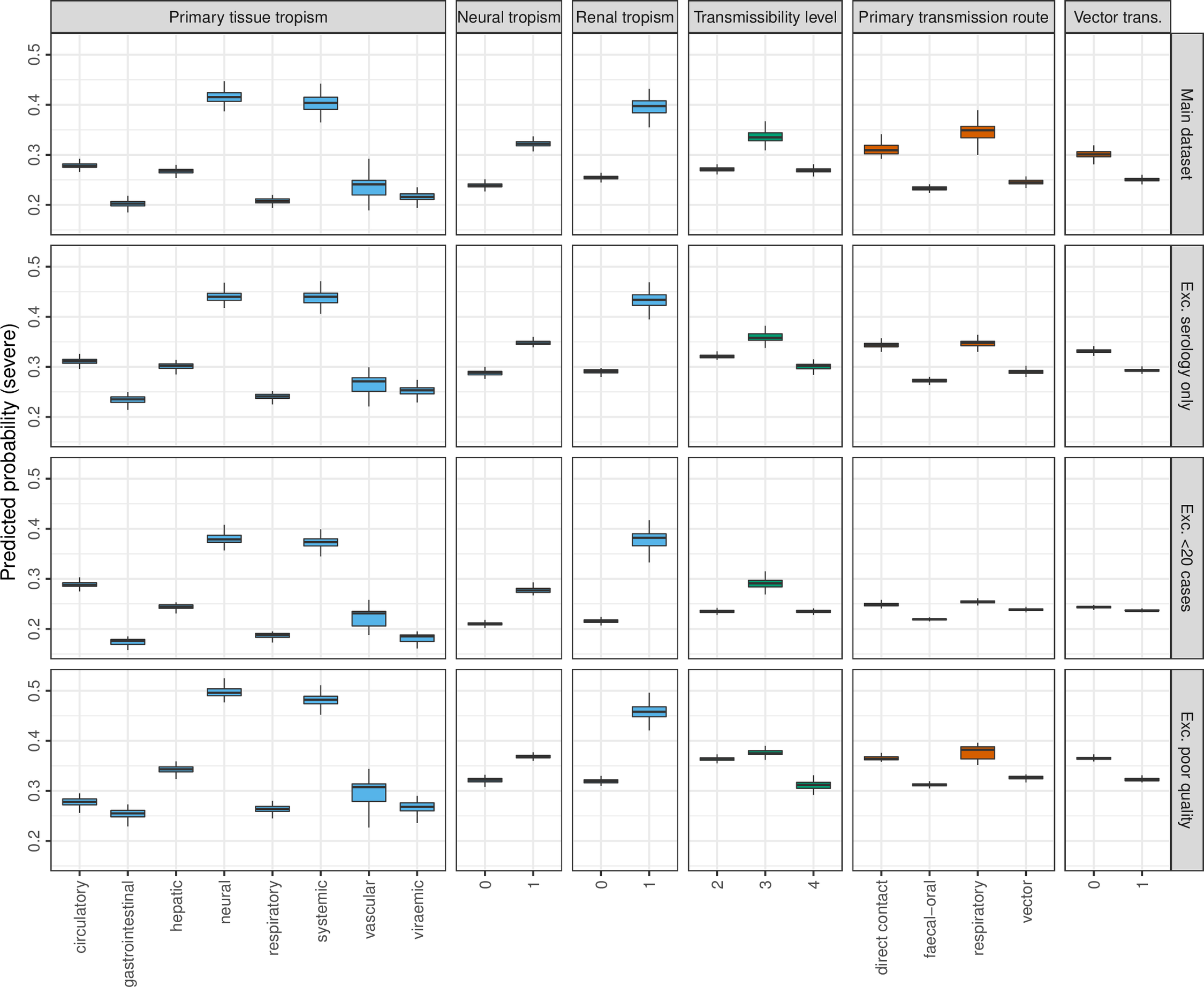

Supplement: S2 Fig — Predicted probability of classifying virulence as ‘severe’ for each of the most informative risk factors from random forest models applied to data sets excluding a) viruses only known to infect humans from serological evidence (n = 36), b) viruses with <20 recognised human infections (n = 55), and c) viruses with poor data quality in at least one predictor (n = 71) alongside predicted probabilities from the main analysis for comparison. Probabilities given are marginal, i.e., averaging over any effects of other predictors. Because each data subset required resampling of the training and test partitions, note that raw prevalence of ‘severe’ virulence differed between each model (see S3 Table). Boxes denote distribution of probabilities across 200 training/test partitions, with heavy lines denoting median probability. Colour key denotes predictor variable type as in Fig 3, i.e., blue = tissue tropism, green = transmissibility, red = transmission route. Supporting data are available via figshare: 10.6084/m9.figshare.7406441.v3 (https://figshare.com/articles/Data_and_supporting_R_script_for_Tissue_Tropism_and_Transmission_Ecology_Predict_Virulence_of_Human_RNA_Viruses/7406441/3). (TIF) [file pbio.3000206.s007.tif]

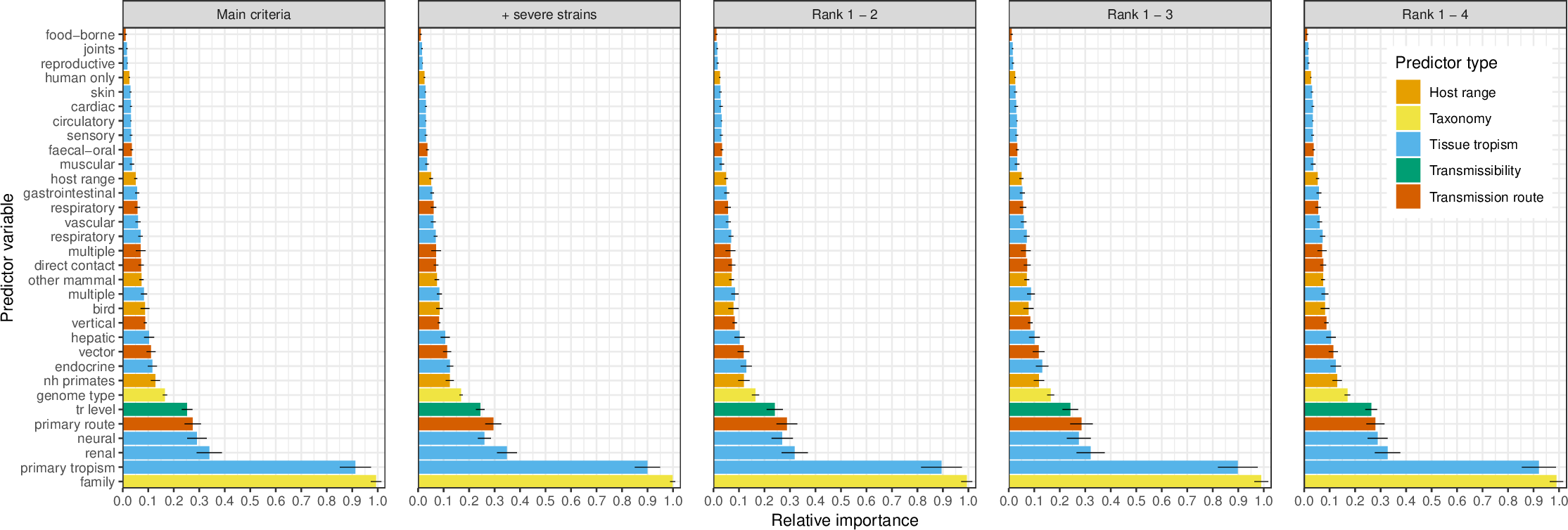

Supplement: S3 Fig — Variable importance for virulence risk factors from random forest models predicting alternative virulence measures using different two-category definitions of ‘severe’, calculated as the relative mean decrease in Gini impurity scaled against the most informative predictor within each model alongside importance from the main analysis for comparison. Points denote mean values across 200 training/test partitions. Error bars denote ± 1 standard deviation. Colour key denotes type of predictor variable. Supporting data are available via figshare: 10.6084/m9.figshare.7406441.v3 (https://figshare.com/articles/Data_and_supporting_R_script_for_Tissue_Tropism_and_Transmission_Ecology_Predict_Virulence_of_Human_RNA_Viruses/7406441/3). (TIF) [file pbio.3000206.s008.tif]

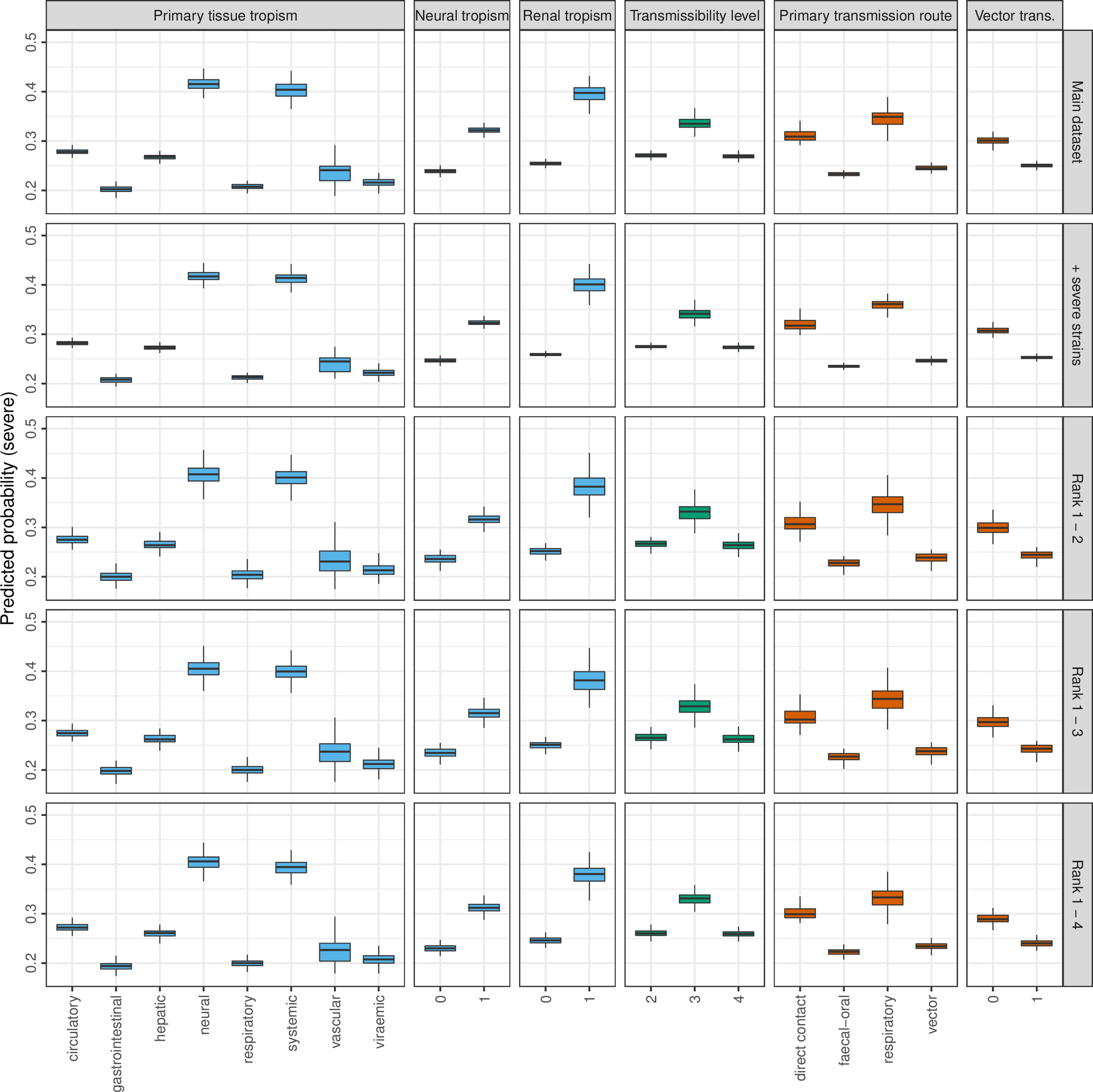

Supplement: S4 Fig — Predicted probability of classifying virulence as ‘severe’ in alternative virulence measures for each of the most informative risk factors from random forest models alongside predicted probabilities from the main analysis for comparison. Probabilities given are marginal, i.e., averaging over any effects of other predictors. Because each measurement used a different two-category definition of ‘severe’, note that the raw prevalence of ‘severe’ virulence differed between each model (see S5 Table). Boxes denote distribution of probabilities across 200 training/test partitions, with heavy lines denoting median probability. Colour key denotes predictor variable type as in Fig 3, i.e., blue = tissue tropism, green = transmissibility, red = transmission route. Supporting data are available via figshare: 10.6084/m9.figshare.7406441.v3 (https://figshare.com/articles/Data_and_supporting_R_script_for_Tissue_Tropism_and_Transmission_Ecology_Predict_Virulence_of_Human_RNA_Viruses/7406441/3). (TIF) [file pbio.3000206.s009.tif]
